# Messinian age and savannah environment of the possible hominin *Graecopithecus* from Europe

**DOI:** 10.1371/journal.pone.0177347

**Published:** 2017-05-22

**Authors:** Madelaine Böhme, Nikolai Spassov, Martin Ebner, Denis Geraads, Latinka Hristova, Uwe Kirscher, Sabine Kötter, Ulf Linnemann, Jérôme Prieto, Socrates Roussiakis, George Theodorou, Gregor Uhlig, Michael Winklhofer

**Affiliations:** 1 Department of Geosciences, Eberhard-Karls-University Tübingen, Germany; 2 Senckenberg Centre for Human Evolution and Palaeoenvironment, Tübingen, Germany; 3 National Museum of Natural History, Bulgarian Academy of Sciences, Sofia, Bulgaria; 4 CR2P (UMR 7207), Sorbonne Universités, Muséum National d'Histoire Naturelle, Centre National de la Recherche Scientifique, CP 38, Paris, France; 5 Department of Human Evolution, Max Planck Institute for Evolutionary Anthropology, Deutscher Platz 6, Leipzig, Germany; 6 Department of Earth and Environmental Sciences, Geophysics, Ludwig-Maximilians-University, Munich, Germany; 7 Senckenberg Natural History Collections Dresden, Museum of Mineralogy and Geology, GeoPlasmaLab, Dresden, Germany; 8 Department of Earth and Environmental Sciences, Palaeontology, Ludwig-Maximilians-University, Munich, Germany; 9 Faculty of Geology and Geoenvironment, National and Kapodistrian University of Athens, Greece; 10 Leibniz Institute of Polymer Research Dresden, Dresden, Germany; 11 School of Mathematics and Science, Institute of Biology and Environmental Science, University of Oldenburg, Germany; Université de Poitiers, FRANCE

## Abstract

Dating fossil hominids and reconstructing their environments is critically important for understanding human evolution. Here we date the potentially oldest hominin, *Graecopithecus freybergi* from Europe and constrain the environmental conditions under which it thrived. For the *Graecopithecus*-bearing Pikermi Formation of Attica/Greece, a saline aeolian dust deposit of North African (Sahara) provenance, we obtain an age of 7.37–7.11 Ma, which is coeval with a dramatic cooling in the Mediterranean region at the Tortonian-Messinian transition. Palaeobotanic proxies demonstrate C4-grass dominated wooded grassland-to-woodland habitats of a savannah biome for the Pikermi Formation. Faunal turnover at the Tortonian-Messinian transition led to the spread of new mammalian taxa along with *Graecopithecus* into Europe. The type mandible of *G*. *freybergi* from Pyrgos (7.175 Ma) and the single tooth (7.24 Ma) from Azmaka (Bulgaria) represent the first hominids of Messinian age from continental Europe. Our results suggest that major splits in the hominid family occurred outside Africa.

## Introduction

The Late Miocene was a time of major hominine radiation (African apes and humans[1]), but when, where, and why lineages split is debated intensely[2, 3]. Recent discoveries[4] with potential hominin (humans and their non-ape ancestors) affinities[5] in Greece (Attica) and Bulgaria (Upper Thrace) raise questions about the age and origin of these candidate pre-humans and the environmental conditions under which they thrived in Europe. Exact dating of *Graecopithecus* and reconstruction of its habitats in Attica and Upper Thrace may, therefore, shed new light on the debate on hominin origins.

**Fig 1:**
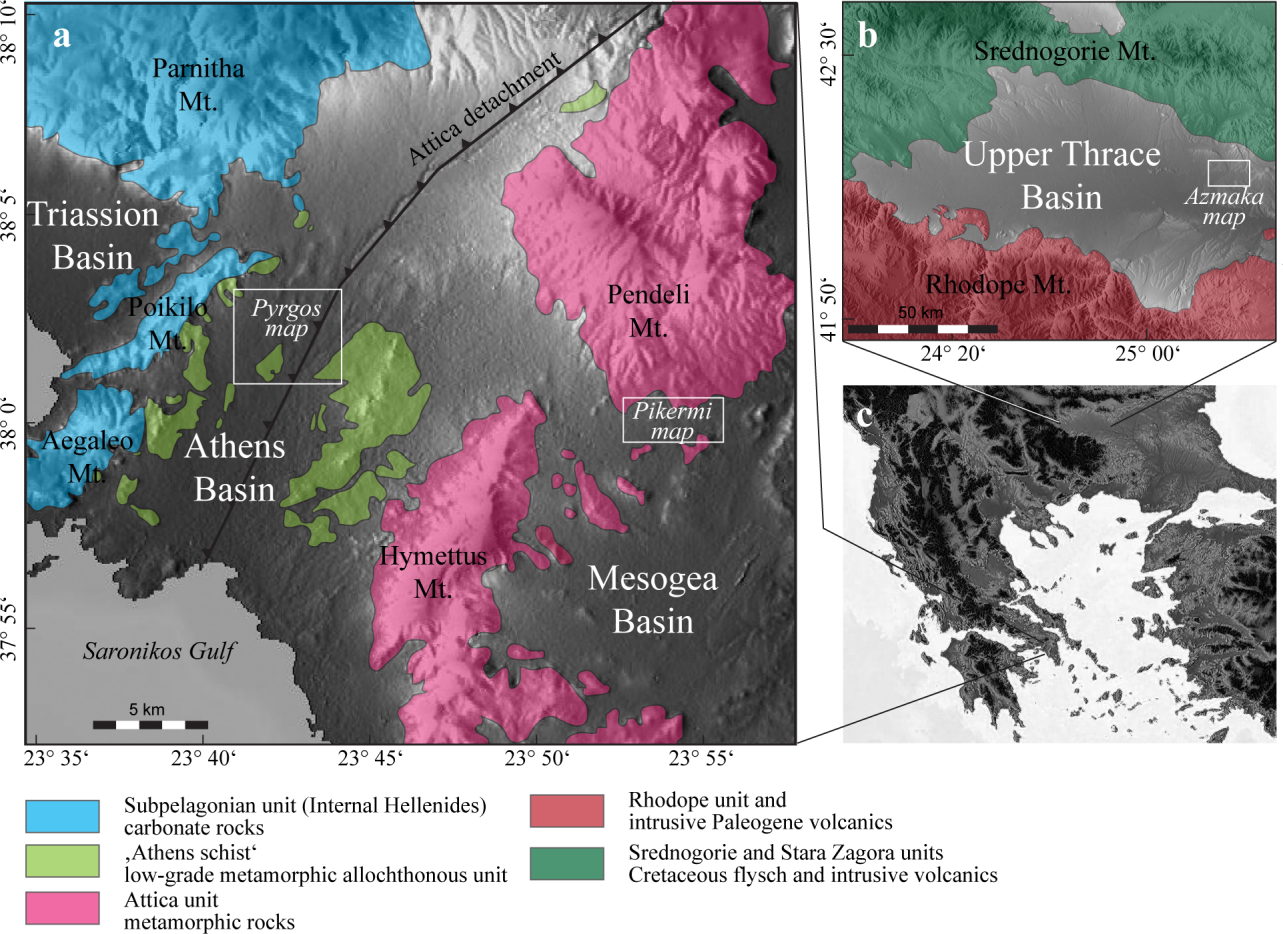
Locations of *Graecopithecus*-bearing exposures. Digital elevation map of **a**, Attica (Greece) and **b**, Upper Thrace (Bulgaria) with main structural units indicated. The positions of the geological maps of Pyrgos Vassilissis, Pikermi and Azmaka (see Fig 2) are indicated. **c**, Digital elevation map of the Balkan Peninsula.

The type mandible of *Graecopithecus freybergi* was found in the Athens Basin of southern Attica near Pyrgos Vassilissis Amalias[6], an area that is today largely overbuilt by the rapidly growing Greek capital. To resolve the site stratigraphy it is necessary to study the adjacent Mesogea Basin, which preserves the famous bone accumulations of Pikermi, which have been excavated for nearly 180 years[7] and are displayed in museums worldwide. Both the Athens and the Mesogea basins developed during the Late Miocene by activation of a major detachment fault[8], which separates carbonates of the Internal Hellenides from Mesozoic metamorphic rocks (Fig 1). The thick continental basin deposits consists of coarse grained alluvial fan sediments (e.g. debris-flows) and palustrine and lacustrine sediments (coal, platy limestones; [9–11]), with deposition starting during the early Tortonian [12]. The second *Graecopithecus* fossil, a single tooth, derives from Upper Miocene sediments at Azmaka in the Upper Thrace Basin[4]. This basin is formed by Neogene extension [13] and is filled by the fluvial Ahmatovo Formation [4, 14–16], at the base of which the hominid tooth was found [4, 16]. Here we reconstruct environmental conditions from the two *Graecopithecus*-bearing sediment successions using grain-texture analysis, end-member modelling of grain-size distributions, geochemistry of soluble salts and provenance analysis of U-Pb ages of detrital zircons. We then provide age constraints on fossils and document environmental changes on the basis of combined bio-magnetostratigraphy and cyclostratigraphy. Furthermore, we analyse vegetation using phytoliths and palynology and discuss changes in large mammal associations to elucidate landscapes and the biogeography of this putative oldest hominin.

## Materials and methods

### Grain-size analysis

To investigate depositional environment processes we determine particle size distributions with a Laser Particle Sizer (Mastersizer 2000, Malvern Instruments, University of Tübingen) using Sodiumpyrophosphate (Na_4_P_2_O_7_ ∙ 10 H_2_O) as a dispersant. Pre-treatment of samples (including decalcification) follows ref.[17].

### Silt grain texture

To characterize transport mechanisms surface micro-texture of quartz grains from several silt samples of the Pikermi Formation from Pyrgos, Pikermi and Chomateri, as well as the Rafina Formation were studied under scanning electron microscope (SEM) at the University of Tübingen. Texture results are interpreted according to refs. [18–21].

### End member modelling of grain-size spectra

End-member analysis was performed to identify sedimentologically distinct components (end members) in the grain-size data set and to study temporal variations of component fractions. The underlying assumption is that measured grain-size distribution variations along the sampled profiles largely represent variations in the physical mixing ratios of a few end-member grain-size distributions (see S1 Text for detailed descriptions).

### Dust mass accumulation rate

From the tie points in the age model, we calculated linear sedimentation rates *sr*_*i*_, from which we obtained the dust mass accumulation rate (DMAR) as:

$${DMAR}_{i}=\rho \;{sr}_{i}\left(\sum_{j=1}^{j_{max}}y_{ij}\right)/\left(\sum_{j=1}^{n}y_{ij}\right)$$

where *ρ* is the typical mineral dust density (2.65 g/cm^3^), *y*_*ij*_ is the *j* grain size bin at the *i*-th sample position, *j*_max_ is the grain size bin corresponding to a maximum grain size of 30 μm, while *n* is the total number of grain size bins to ensure proper normalization of the grain size fraction < = 30 μm.

### Ion chromatography

To determine total soluble salt (TSS) content and ionic composition of silts from the Pikermi Formation we used an ICS 1000 (Dionex) ion chromatograph at the University of Tübingen and measured cations (Li, Na, K, Mg, Ca) and anions (fluoride, chloride, bromide, nitrate, sulfate, phosphate) from 25 leached sediment samples (S1 Table). To characterize the source of TSS we used different ionic and molar ratios. The Ca^2+^/Cl^-^ ionic ratio is an index for predominance of continental or atmospheric (marine-based aerosols) salts. This ratio is 0.02 in modern sea-water and a ratio <1 characterizes marine aerosols, whereas ratios >1 are typical of continental aerosols[22]. The Cl^-^/Br^-^ molar ratio is used to differentiate between marine and ‘continental’ chloride (evaporative salts)[23]. Ratios higher than sea-water (ratio 655 ±4) indicate addition of evaporative salts (halite). Where the Na^+^/Cl^-^ ionic ratio is similar to sea-water (0.56) and the Na^+^/Cl^-^ molar ratio is nearly 1, halite is an additional Cl^-^ source (S1 Table). Higher Na^+^/Cl^-^ ionic ratios than sea-water may be related to additional contributions from non-chloridic Na-salts, or more likely, chloride depletion by photochemical reactions[24].

### U-Th-Pb isotopes

To reveal the source of silt-grain particles, we analyse detritic zircons for U, Th, and Pb isotopes by LA-SF ICP-MS (Laser Ablation combined with Inductively Coupled Plasma Mass Spectronometry) at the Museum für Mineralogie und Geologie (GeoPlasma Lab, Senckenberg Naturhistorische Sammlungen Dresden), using a Thermo-Scientific Element 2 XR sector field ICP-MS coupled to a New Wave UP-193 Excimer Laser System. A teardrop-shaped, low volume laser cell was used to enable sequential sampling of heterogeneous grains (e.g., growth zones) during time resolved data acquisition (see S2 Text for further details).

### Fossil collections and fieldwork

The Pyrgos Vassilissis vertebrate fossils are deposited in the Naturhistorische Gesellschaft Nürnberg (v. Freyberg collection numbers TE 101–133) and the Palaeontological Museum University of Athens (Paraskevaidis collection, prefix AMPG). No permits for geologic fieldwork in Azmaka (Bulgaria) and Pikermi (Greece) were required for the described study.

### Magnetostratigraphy

To generate a magnetostratigraphic record a total of 118 oriented samples were taken from the Pikermi (92), Rafina (14), and Ahmatovo (12) formations. In Pikermi, six spatially separated sections were sampled: Chomateri-A (abbreviation: Chom A, 22 samples), Chomateri-B (Chom B, 22 samples), Pikermi-A (PV1, 22 samples), Pikermi-B (PV3, 24 samples) within the Pikermi Formation and Rafina-A (Raf, 8 samples) and Rafina-B (Raf2, 6 samples) from the top of the overlying Rafina Formation. Orientations of the samples and bedding planes were determined using a Brunton compass. Additionally, two 25-mm core samples were drilled from the consolidated sediment infill of two giraffid long-bones from Pyrgos. The sediment of these two bones (TE 124, 130) represents the only available primary deposits from the Pyrgos section. The palaeo horizon within these bones is defined by geopetal structures (S1 Fig). Due to these characteristics, only the top and bottom of the sample is known, whereas the azimuth is unknown. Therefore, only the inclination of the resulting magnetic signal is meaningful and yields the magnetic polarity during deposition. A first set of 50% of the samples, including all lithologies and formations, was subjected to alternating field (AF) demagnetization using the automated system at the Ludwig-Maximillians-University (LMU) Munich, Germany[25] with peak fields of 90 mT. AF demagnetization failed on all haematite-bearing, mainly red sediment samples from the Pikermi and Pyrgos formations. All samples from these formations and the remaining samples from all other formations (Rafina and Ahmatovo) were subjected to thermal demagnetization experiments using a Schonstedt thermal demagnetizer and a 2-G Enterprises Superconducting Rock magnetometer within a shielded laboratory at LMU with peak temperatures of 680°C. Resulting demagnetization data were analysed using principal component analysis[26], using data from at least four consecutive demagnetization steps to define a magnetization component. After distinguishing the characteristic component of the remanent magnetization, the palaeomagnetic direction for each sample was transferred into a virtual geomagnetic pole (VGP). The VGP latitude was used to develop a magnetic polarity pattern for each section.

### Orbital tuning and astrochronology

For calibration we use the bio-magnetostratigraphic age constraints given by the Astronomically Tuned Neogene Time Scale (ATNTS2012,[27]) tuned to insolation seasonality at 40°N (*I*_*40°N 21June*_*−I*_*40°N 21Dec*_ of the astronomical solution La04 with present-day values for the dynamical ellipticity of the Earth and tidal dissipation by the moon[28]). We use this insolation curve rather than the similar 65°N summer insolation and the summer inter-tropical insolation gradient (SITIG), because it appears more appropriate for the Mediterranean. High seasonal insolation contrast during precession minima and obliquity maxima has been attributed to increased Mediterranean winter rainfall[29] related to convective precipitation[30].

Fluvial runoff and debris-flow occurrence are accelerated during times of increased seasonal precipitation[31, 32], which is why we chose for orbital calibration to tune the mid-points of fluvial channel-trains (Chomateri Member) and debris flows (Red Conglomeratic Member) to insolation seasonality maxima. Our orbital tuning of the Pikermi Formation suggests that between sub-sections PV3 and PV1 less than a precession cycle is missing in our stratigraphic record.

### Phytoliths

To reconstruct vegetation we use the phytolith approach. Phytolith identification follows strictly the codes and standards of the International Code for Phytolith Nomenclature (ICPN)[33]. For ambiguous phytolith nomenclature and classification we use the PHYTCORE database (www.gepeg.org/cercador.asp) [gepeg]. We analyse 10 samples (smear slides) from the Pikermi Formation (including the Pyrgos fossiliferous level) and one sample from the Ahmatovo Formation (Azmaka), where we consider all phytolith size fractions (usually <100 μm). For details on phytolith taxonomy and applied phytolith indices see S3 Text.

### Palynology and micro-charcoal

To supplement the reconstruction of vegetation and to investigate potential signals of fire ecology we analyze pollen and micro-charcoal content. Ten samples from the Pikermi Formation were analysed for palynomorphs and charcoal. For segregation of the organic fraction we used the following method: 5 g of dry material from every sample was homogenized, suspended in 100 ml of distilled water and decalcified with 100 ml of 30% HCl. For quantification of organic particles we added *Lycopodium* marker tablets to the suspension (Batch number 938934, Lund University with a mean spore concentration of 10,679). The palynomorph containing grain size fraction was concentrated by sieving between 125 μm and 6 μm. The light organic fraction was segregated from heavy mineral components by density fractionation using saturated ZnCl solution for 5 min at 1000 rpm. The supernatant was washed with distilled water and was mounted on microscope slides with glycerine gelatine. Palynomorphs and charcoal particles (CP) were identified and counted at 400x magnification with an Olympus BX50 light microscope with an attached digital camera. Quantification was accomplished by alignment with the *Lycopodium* standard. *Lycopodium* marker-spores have been counted to approximately 100 in every sample. For determination of palynomorphs we used ref. [34] and [35].

### Stable isotopes of pedogenic carbonates

To support our environmental reconstructions we investigate stable isotope composition of pedogenic carbonate. Carbon and oxygen isotopes of pedogenic carbonates from three samples from the classical Pikermi level (PV3) and 12 samples from the *Graecopithecus*-level of Pyrgos were analysed at the University of Tübingen with a GasBench II connected to a mass spectrometer (Finnigan Mat 252) via continuous flow. Calibration standards are NBS18 (δ^13^C = -5.00, δ^18^O = -22.96 ‰, relative to VPDB) and NBS19 (δ^13^C = 1.95, δ^18^O = -2.20 ‰, relative to VPDB), with a reproducibility of ±0.1‰ for δ^13^C and ±0.1‰ for δ^18^O analyses. The external reproducibility for carbonate content is ±10%. Acidic fractionation is determined for calcite.

## Results and discussion

### Sedimentology and lithostratigraphy

#### Athens and Mesogea Basin

**Fig 2:**
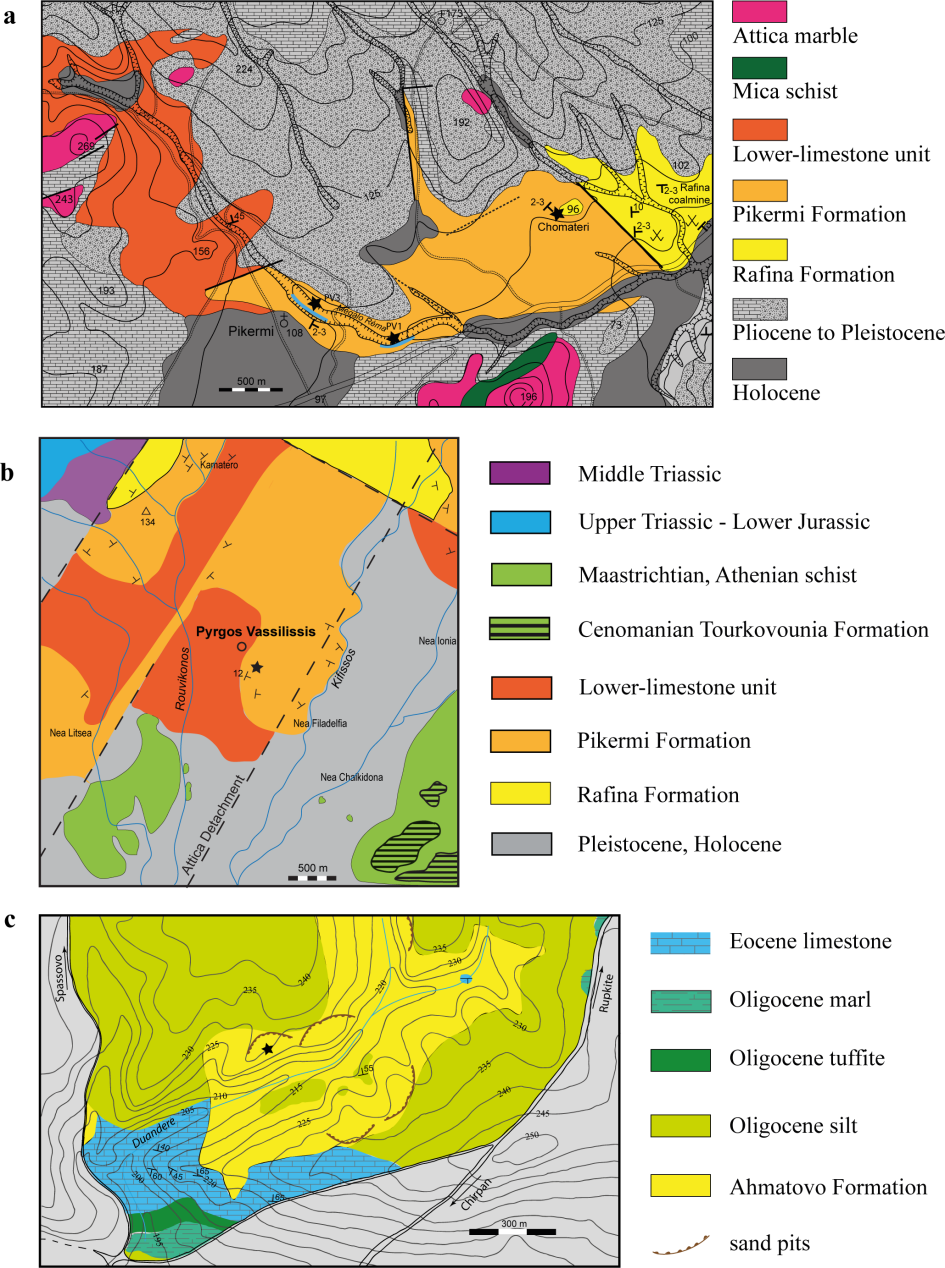
Geological maps of the regions around the studied localities. **a**, Map of the Pikermi area (Mesogea Basin, Attica, Greece) modified after ref. 31. Sampling points (black stars). PV3 –Pikermi valley 3 (new excavation of Theodorou 2010 = old excavation of Gaudry 1855–1860, Woodward and Skoufos 1901, Abel 1911–1912), PV1 –Pikermi valley 1 (new excavation of Theodorou 2009), Chomateri (old excavations of Symeonidis & Bachmayer 1972–1980). Blue lines represent measured sections along the Megálo Réma rivulet. **b**, Map of the Pyrgos Vassilissis area (Athens Basin, Attica, Greece) modified after ref. 28, 32. The position of the type locality of *Graecopithecus freybergi* is indicated by a black star. **c**, Map of the Azmaka area (3 km north of Chirpan, Upper Thrace Basin, Bulgaria). The location where the *Graecopithecus* tooth was found in the Azmaka quarry is indicated by a black star.

**Fig 3:**
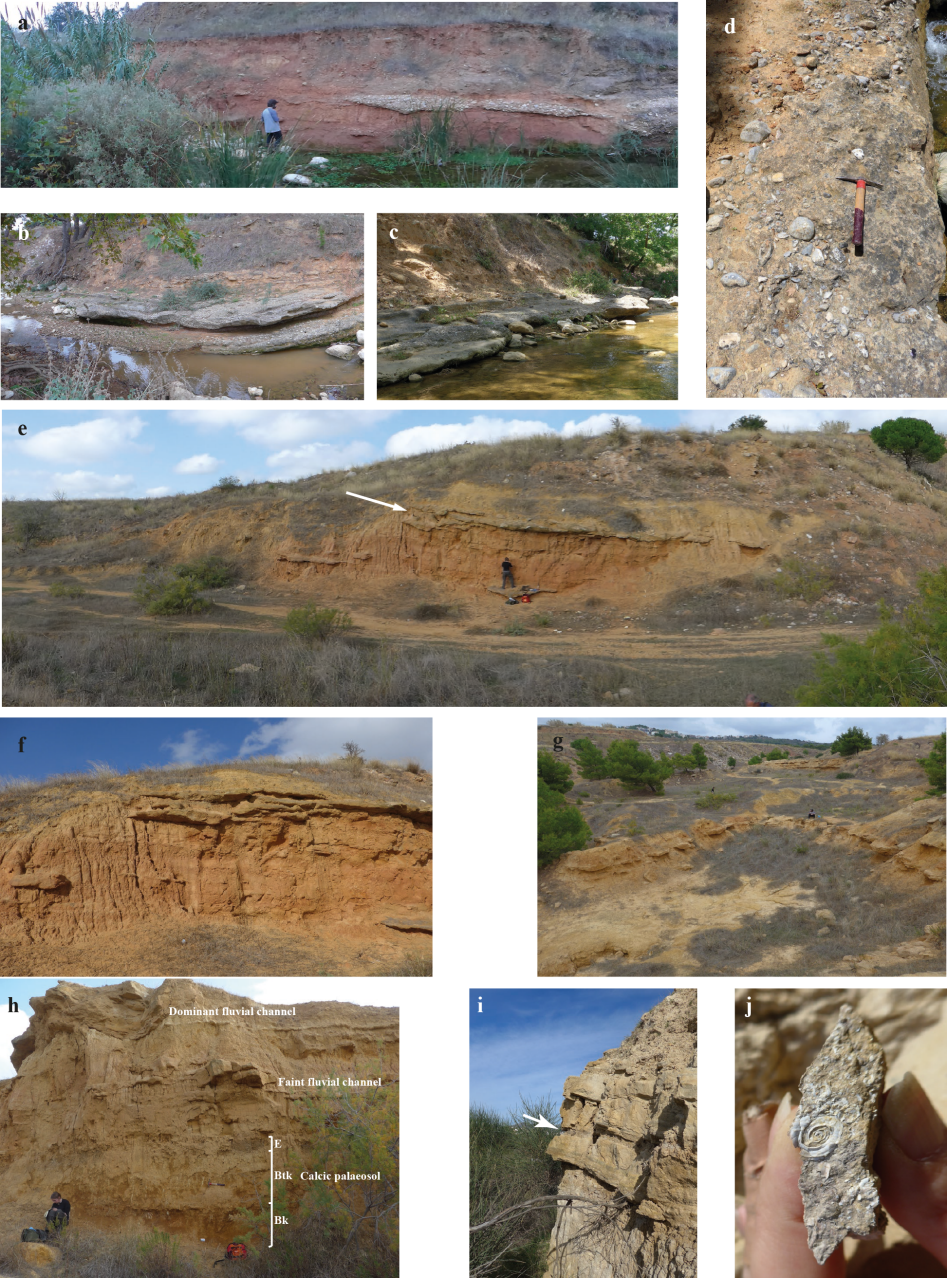
Field photographs of the Pikermi and Rafina formations. **a-d**, Red Conglomeratic Member of the Pikermi Formation along the Megálo Réma rivulet. **a**, Red aeolian silts and conglomeratic levee of a dominant debris-flow from the upper part of the PV1 section. **b**, Lenticular shaped, dominant (‘doubled’) debris-flow, middle of PV3 section. **c**, Same debris-flow as b with undulating surface. **d**, Same debris-flow as b and c, with projecting cobbles (length of hammer 35 cm). **e-g**, Transition from the Red Conglomeratic Member to the Chomateri Member of the Pikermi Formation in the northern former clay pit Chomateri. **e**, Section Chom-A (northern view) with slight dip of sediments to the south-east. The transition between members is indicated by an arrow. **f**, Same as e, with channel-fill trains at the base of the Chomateri Member. **g**, view to the east of laterally continuous channel-fill trains. **h**, Chomateri Member of the Pikermi Formation in the southern former clay pit Chomateri. Section Chom-B showing the prominent 2.2-m-thick calcic palaeosol (E-Btk-Bk soil horizons) at the base (Btk and Bk horizons deeply rooted by macro-rhizolithes), overlain by faint and dominant fluvial channel-fill trains. **i, j**, Upper part of the Rafina Formation. **i**, Lacustrine marls and limestones with organic-rich interlayers (arrow). **j**, *Planorbis* shells from marl horizons.

**Fig 4:**
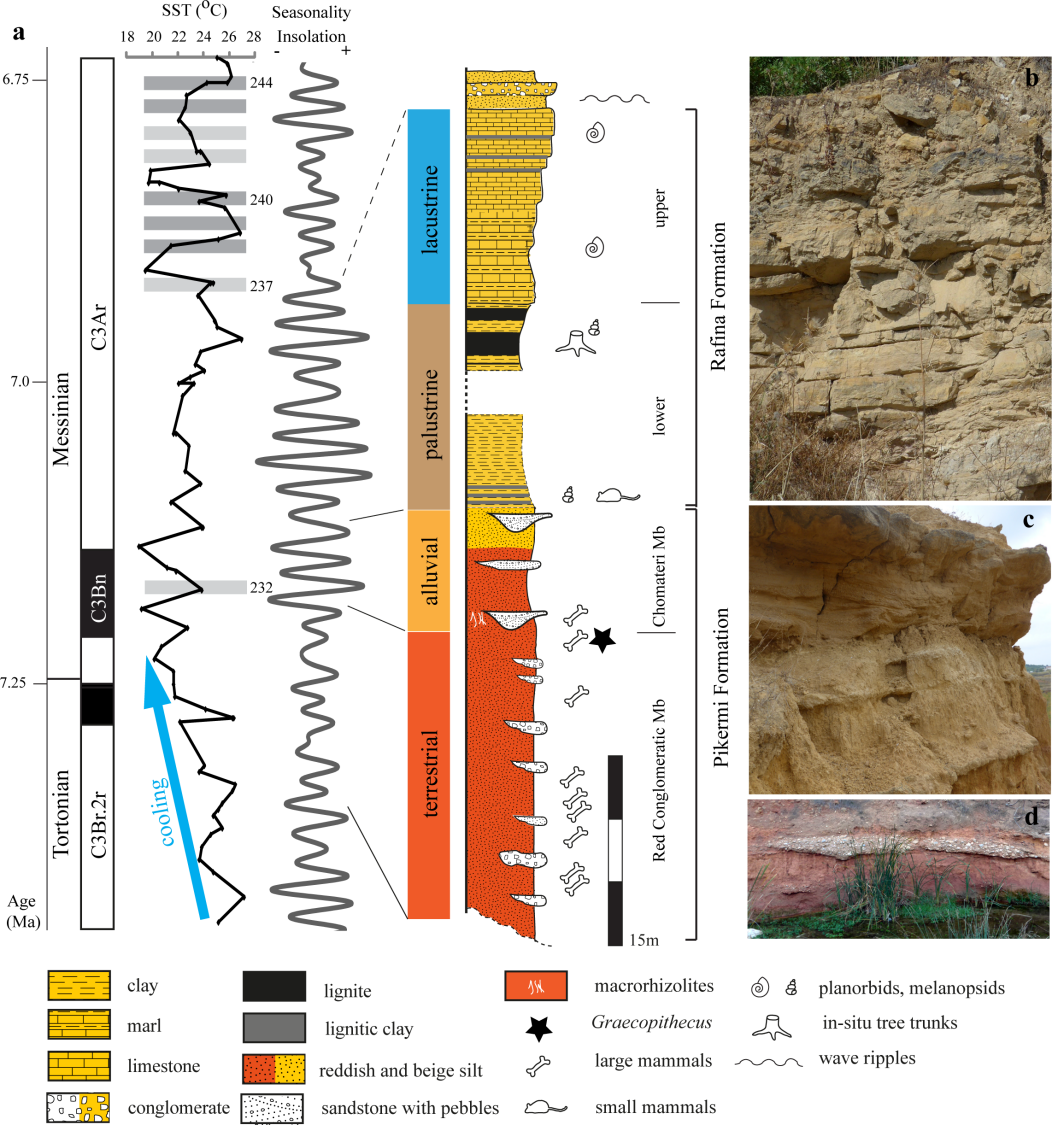
Upper Miocene sediments of southern Attica. **a,** Simplified stratigraphic column of the Pikermi and Rafina formations (profile of the Rafina Formation according to[40]), sedimentary facies development, and correlation to chronostratigraphy, alkenone-based eastern Mediterranean Sea Surface Temperatures[41], and insolation seasonality at 40°N[28]. Grey bars and numbers represent Mediterranean sapropel layers (dark grey–prominent sapropel, light grey–distinct sapropel) of the Tortonian type section at Monte dei Corvi[42]. Blue arrow indicates intense cooling during the latest Tortonian. **b-d**, Typical outcrop views of the upper Rafina Formation platy limestones (b, height of image = 1.5 m), alluvial sandstones of the Chomateri Member (c, height of image = 1 m), and red silt with debris-flow of the Red Conglomeratic Member (d, height of image = 2 m). For further details see Fig 3.

We subdivide Upper Miocene sediments of the Athens and the Mesogea Basins (Figs 1 and 2) into the terrestrial to alluvial *Pikermi Formation* (new formation; see S4 Text for descriptions) and the palustrine to lacustrine *Rafina Formation* (new formation; see S4 Text for descriptions). The Pikermi Formation represents an up to 30-m-thick sequence of predominantly reddish silts with subordinate clastic channels of conglomerates and sandstones, which contains a rich and exclusively terrestrial vertebrate fauna. The formation rests discordantly upon the ‘lower limestone unit’[9] (palustrine to lacustrine grey marls and coals) and is concordantly overlain by the Rafina Formation (palustrine to lacustrine clay, coal, and platy limestone). Based on transport mechanisms, sediment colour, and palaeosol development, the Pikermi Formation can be subdivided into two members: the Red Conglomeratic Member (new member; see S4 Text for descriptions) characterized by debris flows and the fluvio-alluvial Chomateri Member (new member; see S4 Text for descriptions). The lower part of the Pikermi Formation (Red Conglomeratic Member) represents an alternation of red silts with a weak pedogenic overprint and debris flow deposits (Fig 3). These debris flows contain clasts of the nearby Attica unit of Mt. Pendeli, which indicates a strong topographic gradient. Silts from the lower Red Conglomeratic Member include the classical Pikermian bone accumulations[36]. The upper Pikermi Formation (Chomateri Member) represents an alternation of reddish to yellowish silts with fluvial channels and channel-fill trains (Fig 3) that are indicative of small migrating streams during times of increased surface run-off. Away from channels, silts can contain well developed calcareous palaeosols rich in mammalian fossils[37]. In the Athens Basin the Pikermi Formation is best known from the Pyrgos outcrop ([6, 9]; Fig 2). In both basins, the Pikermi Formation is concordantly overlain by palustrine clays and coals, and lacustrine marls and limestones of the Rafina Formation (Fig 4). The type mandible of *Graecopithecus freybergi* was found in Pyrgos Vassilissis at the top of the Red Conglomeratic Member. The 30-to-35-m thick Rafina Formation can be subdivided into a lower palustrine part and an upper lacustrine part (see[38, 39] and S4 Text).

#### Upper Thrace Basin

Late Miocene fluvial sedimentation characterizes the Upper Thrace Basin. These up to 300-m-thick clastic sediments belong to the *Ahmatovo Formation*[14], which are attributed to initiation of the palaeo-Maritsa drainage[15]. The base of the Ahmatovo Formation crops out in abandoned sand quarries near Azmaka, 3.5 km north of the city of Chirpan (Fig 2C,[4]). The 26 m composite stratigraphy represents a stacked sequence of six fining-upward cycles. Alternations of cobbly gravels and sand with fine-clastic overbank sediments are interpreted to have been deposited in braided and meandering rivers[16]. Overbank deposits, composed of greenish to yellowish clayey and fine-sandy silts, have been subjected to variable pedogenesis. The *Graecopithecus* tooth was recovered from the fourth cycle, but large mammal fossils are found scattered throughout the profile, both in coarse and fine clastic sediments. The single observed fossil accumulation (Azmaka 6), which produces the bulk of Azmaka fauna, occurs on top of the section in palaeosols of the last cycle[16].

### Palaeomagnetism

**Fig 5:**
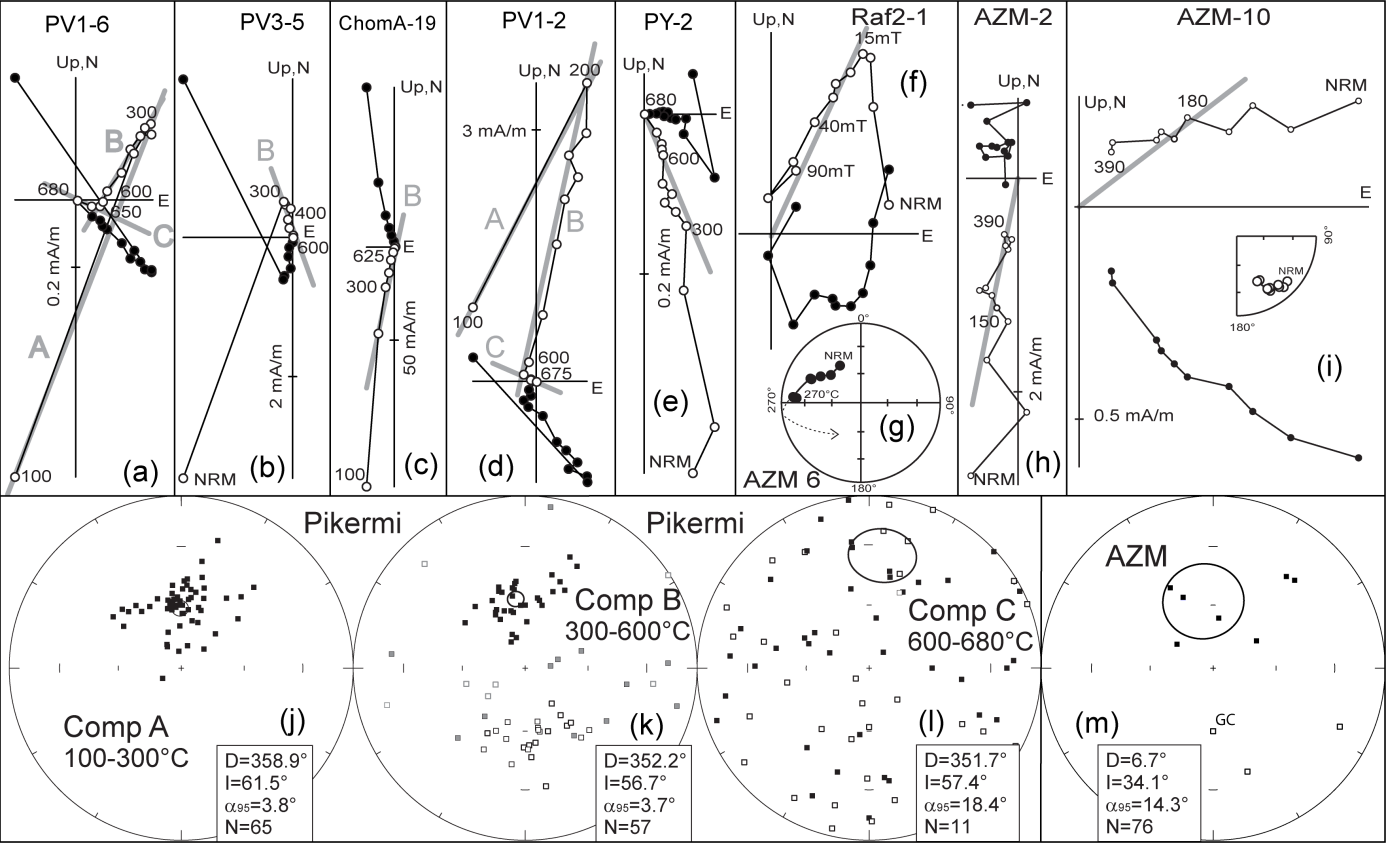
Palaeomagnetic results from each studied section. **a-i**, Representative results of stepwise thermal (alternating field) demagnetization experiments on orthogonal vector endpoint diagrams[44] for samples from the (**a-e**) Pikermi, (**f**) Rafina and (**g-i**) Ahmatovo formations. Open (closed) symbols represent projection onto the vertical (horizontal) plane. **g**, Great circle trend of demagnetization results. Grey lines indicate proposed linear fits of the respective component (component name in grey letters). Steps are in °C (except **f**, where steps are in milliTesla). **j-m**, Stereographic equal-area projections of component mean directions of (**j-l**) Pikermi and (**m**) Ahmatovo formations for indicated components (Pikermi). Overall mean directions (with 95% confidence intervals) are also shown except for (**l**) component C. **k**, Grey marked samples are excluded from the reversals test. **l** Distribution of directions for component C fails a test of randomness[45], where R is lower than R_0_, which indicates a random distribution. **m**, GC indicates that the direction was obtained from the intersection of two remagnetization great circles.

Generally, most samples show well defined behaviour during demagnetization experiments. It was possible to interpret most of them either using linear trends or remagnetization great circles at projected demagnetization steps. Only occasionally, including AF demagnetization of red sediments, demagnetization diagrams did not yield any interpretable magnetic polarities. In contrary, the limestones of the Rafina formation hold a much weaker magnetic signal and the success rate there was only ~40%. All other formations had a moderately high success rate: Chom A: 100%, Chom B: 78%, PV1: 69%, PV3: 64%. Furthermore, palaeomagnetic results can be divided into two groups. Samples from the Ahmatovo and Rafina formations have a weak magnetic signal with mostly only one magnetization component (Fig 5F, 5H and 5I). Three samples had such a strong present-day field overprint that great circles analysis was used to isolate the characteristic magnetization direction ([43]; Fig 5G). The remaining samples from the Pikermi Formation have a strong magnetic signal and thermal demagnetization was necessary to isolate the characteristic remanent magnetization (Fig 5A–5E).

#### Ahmatovo Formation

**Fig 6:**
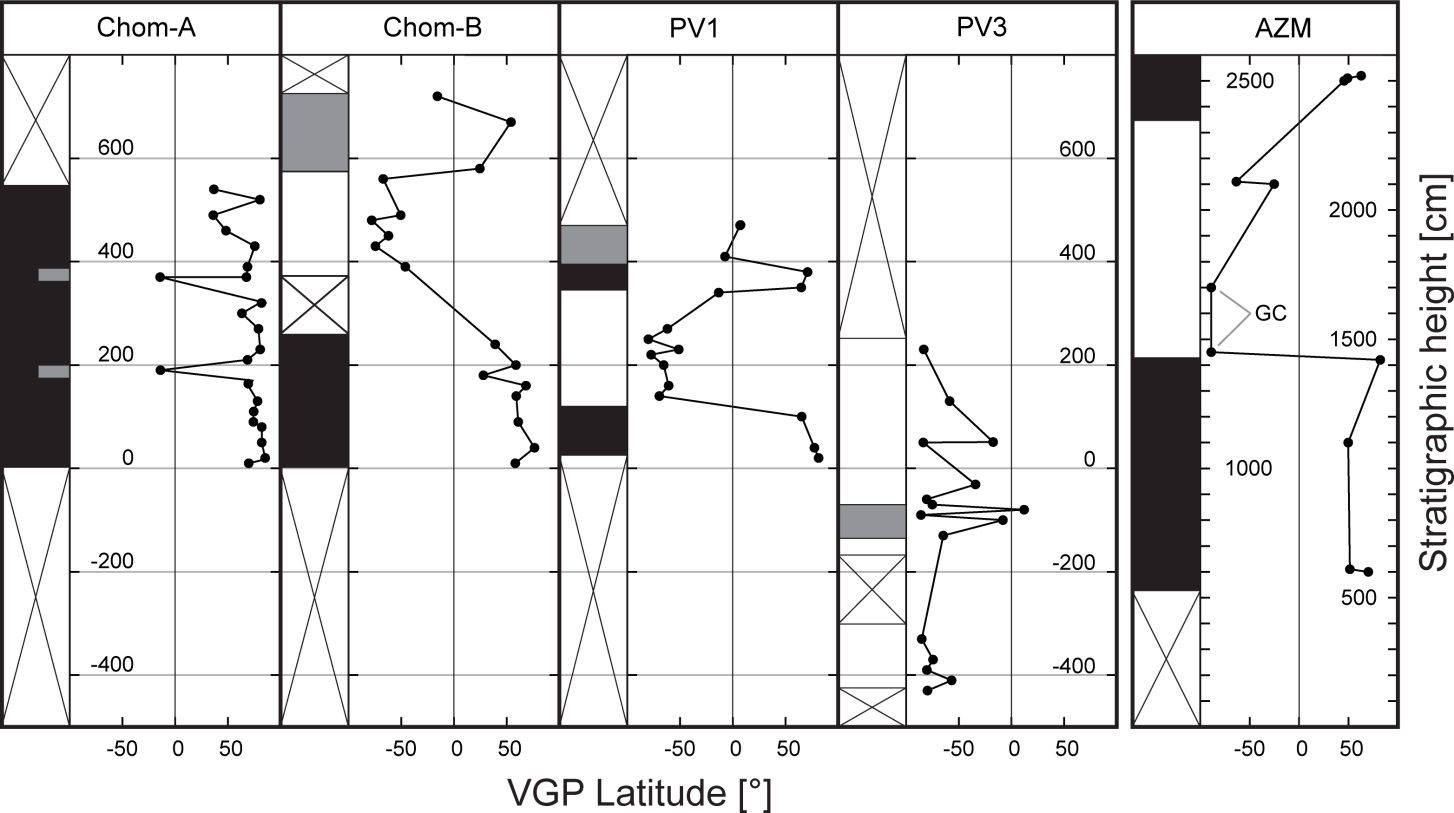
Plot of virtual geomagnetic pole (VGP) latitude versus stratigraphic height for the Pikermi sections and the Ahmatovo section (AZM). The column to the left of each VGP latitude plot indicates the polarity interpretation, where black (white) corresponds to normal (reversed) polarity. Grey areas indicate intervals with uncertain polarity. Crossed intervals were not sampled. GC represents the VGP latitude obtained from the intersection of two remagnetization great circles.

In Azmaka the characteristic component is identified between ~200°C and up to ~400°C and between 20 and 90 mT (Fig 5H and 5I). Three samples, from profile depths between 14 and 18 m, have linear demagnetization trends that clearly miss the origin of the vector endpoint projection. The great circle trend, however, suggests a reversed polarity of the characteristic component for these samples (Fig 5G). Combining all magnetostratigraphic data indicates a reversed polarity interval between profile depths of 14.5 and 23 m, which is surrounded by normal polarity zones (Fig 6).

#### Rafina Formation

Samples from Raf2 have only reversed polarities (Fig 5F), whereas results from Raf are uninterpretable. The latter are chaotically distributed, for which local folds might be responsible.

#### Pikermi Formation

Demagnetization data from these samples are more difficult to interpret. Up to three distinguishable magnetic components are present between 100 and 300°C, between 300 and 600°C, and between 600 and 680°C, and are labelled as components A, B, and C, respectively (Fig 5). Component A has only normal polarities and is related to a present-day field overprint. Component B has a dual polarity pattern and passes the reversal test (classified B after[46], Fig 5K). Component C has a chaotic random distribution of directions (Fig 5J–5L). We interpret these data as resulting from the presence of primary detrital magnetite and secondary hematite. Therefore, we used component B results for magnetostratigraphic analysis. Component C might be explained by a coarse-grained aeolian specularite, whose magnetic directions are strongly influenced by gravitational forces during deposition. Confirmation of this interpretation would require a more detailed rock magnetic study. In terms of the polarity pattern, Chom A has only normal polarity, Chom B has a normal polarity zone overlain by a reversed polarity zone, PV1 contains a reversed polarity zone in the middle surrounded by normal polarity intervals and PV3 has dominantly reversed polarity (Fig 6). Both samples from Pyrgos have positive palaeomagnetic inclinations (Fig 5E), which indicate normal polarity.

### Biochronology and biogeography of Pyrgos Vassilissis mammals

**Fig 7:**
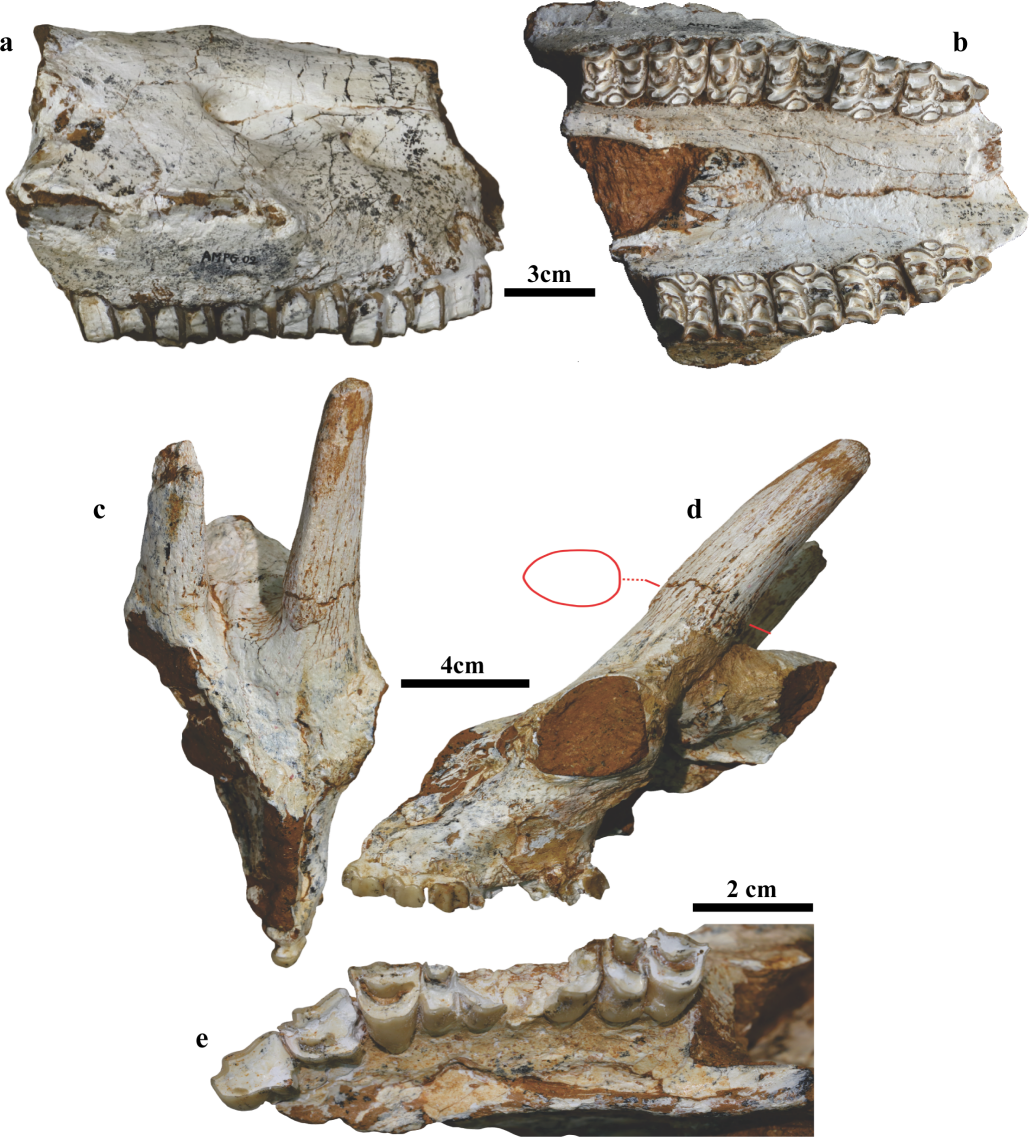
Mammalian fossils from Pyrgos Vassilissis. **a, b,** *Hippotherium brachypus* skull fragment (AMPG 2). **a**, Right lateral view; **b**, ventral view. **c-e**, *Tragoportax macedoniensis*, skull of female individual (AMPG 19a). **c**, Dorsal view; **d**, left lateral view (the red outline indicates the basal cross-section of the left horn-core); **e**, occlusal view of the left toothrow.

The mammalian fauna from Pyrgos (Fig 7, Table 1; see S5 Text and S6–S11 Tables for detailed descriptions and measurements) is generally similar to those of other Turolian sites of the Aegean region. However, several features of the fauna differ from those of the geographically nearby middle Turolian Pikermi site as follows: 1) in *Hippotherium brachypus*, a small preorbital fossa and wide preorbital bar are previously unknown in this species; 2) the presence of a large-sized *Palaeotragus* giraffid; 3) the presence of a large and hypsodont bovid; 4) possibly also the presence of a different rhinocerotid species; and most importantly 5) the presence of the boselaphin bovid *Tragoportax macedoniensis*. Given the small numbers of Pyrgos fossils (n = 49), these differences rule out the contemporaneity of Pyrgos Vassilissis and Pikermi. The presence of *T*. *macedoniensis*, known from the late Turolian (MN 13) faunas of Dytiko, indicates a post-Pikermian age for the *Graecopithecus* faunal assemblage from Pyrgos. Post-Pikermian faunas have been described previously by ref.[4] from the other *Graecopithecus*-bearing locality of Azmaka. From here the first European representatives of the elephantoid *Anancus* are known, which have also been recently described from the Chomateri Member, level Chomateri 1[47]. *Anancus* sp., which characterizes post-Pikermian Late Miocene faunas in the Balkans, is a more plesiomorphic form than the Late Miocene African species [48] and is interpreted as an Asian immigrant[49]. Similarly, the above mentioned Pyrgos mammals unknown from Pikermi (Table 1) could have affinities to Asian taxa. They possibly also have Western Asian/Eastern Mediterranean origins, and might document a new dispersal wave from the east toward the Balkans.

**Table 1:** Mammal taxa recovered from Pyrgos Vassilissis.

Primates	Giraffidae
*Graecopithecus freybergi*	*cf*. *Palaeotragus* sp. large
Carnivora	*Bohlinia attica*
*Adcrocuta eximia*	Bovidae
Proboscidea	*Gazella* sp.
Proboscidea indet.	*Tragoportax* cf. *T*. *amalthea*
Rhinocerotidae	*Tragoportax macedoniensis*
? *Ceratotherium neumayri*	Bovidae sp. large
Equidae	
*Hippotherium brachypus*	

Grey shaded taxa are unknown (or different) from Pikermi and belong to the post-Pikermi fauna.

### Age constraints

**Fig 8:**
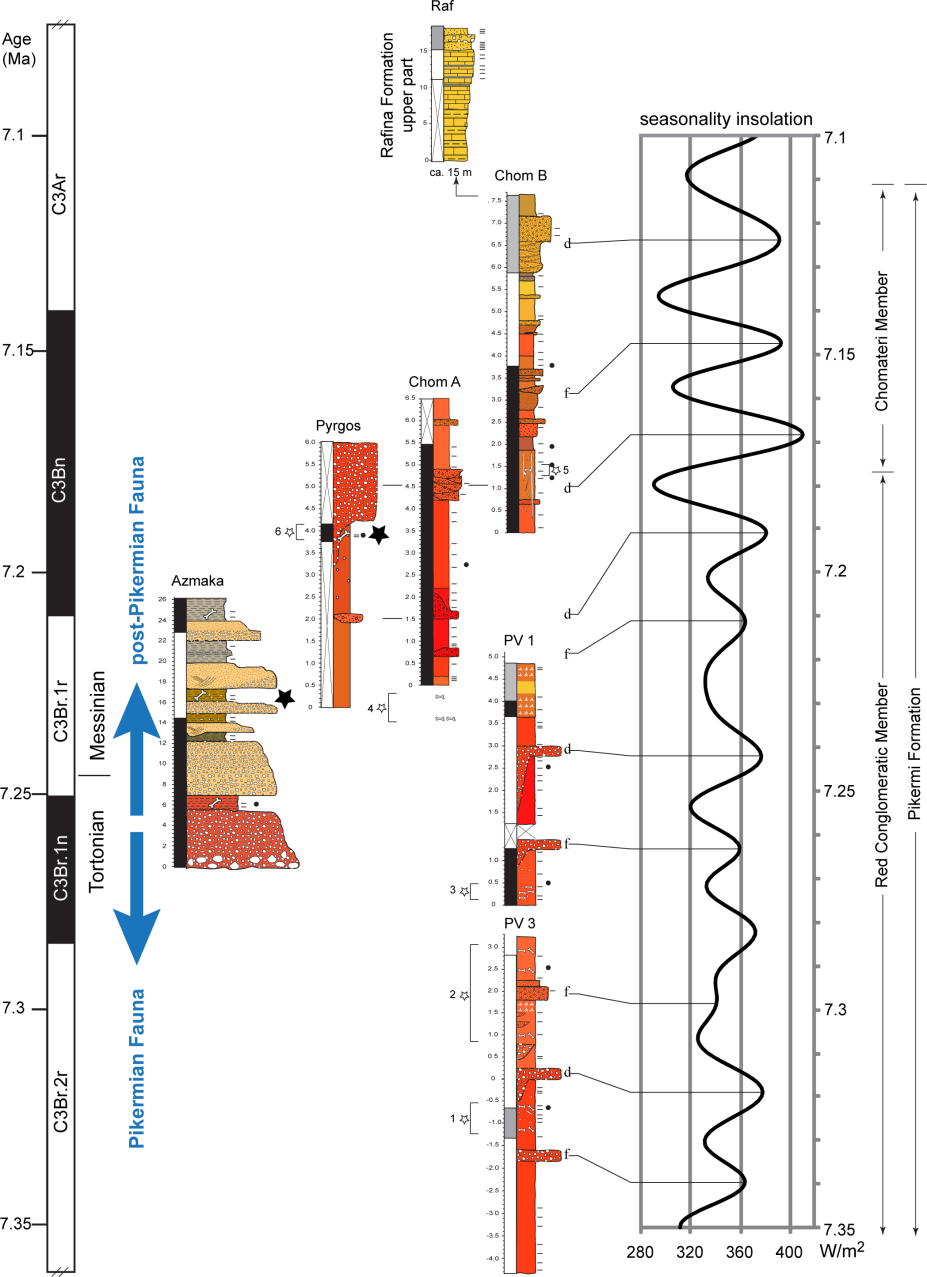
Stratigraphy and chronology of Upper Miocene sections from Attica and Upper Thrace. Bio-magnetostragraphic correlation of Upper Miocene sections from Attica (Pyrgos Vassilissis[9], Chomateri A and B, Pikermi Valley 1 and 3, Rafina) and Upper Thrace (Azmaka) and astronomical tuning of the Pikermi Formation to insolation seasonality at 40°N (*I*_*40°N 21June*_*−I*_*40°N 21Dec*_ of the astronomical solution La04[28]). The orbitally tuned dominant (d) and faint (f) debris flows and fluvial channels are indicated. Black stars represent *Graecopithecus*-levels, dashes (small dots) indicate palaeomagnetic (palaeobotanic) sampling horizons and numbered stars denote horizons of fossil excavations within the Pikermi Formation undertaken by major museums or collections (1 and 2 represent the classical Pikermi levels), 1 –Gaudry 1855 (partim), 1860 (Muséum National d'Histoire Naturelle, Paris), Abel and Skoufos 1912 (Naturhistorisches Museum Wien, University of Athens), 2 –Gaudry 1855, Woodward and Skoufos 1901 (British Museum of Natural History, London, University of Athens), 3 –Theodorou 2009 ff. (University of Athens), 4 –Symeonidis & Bachmayer 1972–1978 (University of Athens, Naturhistorisches Museum Wien), 5 –Symeonidis & Bachmayer 1979–1980 (University of Athens, Naturhistorisches Museum Wien), 6 –v. Freyberg, Paraskevaidis 1944 ff. (University of Erlangen, University of Athens).

Biochronological age constraints for the Pikermi and Ahmatovo formations are derived from their exceptionally rich large mammal record, documented from more than ten accumulation horizons (Fig 8, see S4 Text for details). The middle Turolian fauna of the classical Pikermi levels[36] is considered to be close in age to the Tortonian-Messinian boundary[50]. In contrast, the mammalian faunas of Azmaka, Chomateri-1, and Pyrgos imply a post-Pikermi age because they contain new immigrants such as the proboscidian *Anancus* (for the former two localities) and higher evolutionary stages in several ungulate lineages compared to the classical Pikermi fauna[4]. In particular, most of the mammal taxa of Pyrgos are different from Pikermi (S5 Text; Table 1). Beside a different giraffe species, a new grazing bovid appears, and the hipparion *Hippotherium brachypus* is morphologically distinct from those at Pikermi. Most importantly, the boselaphine bovid *Tragoportax macedoniensis* (Fig 7) links Pyrgos with younger (Messinian) localities of the Balkans. The post-Pikermian newcomers have no discernible affinities to African faunas; their biogeographic relationships are inferred to be eastern Mediterranean.

To constrain the age of *Graecopithecus*-bearing sediments we determined the pattern of palaeomagnetic field directions from seven sub-sections of the Pikermi, Rafina, and Ahmatovo formations, and from Pyrgos. These magnetostratigraphic investigations reveal a distinct pattern of two normal and three (one) reversed polarity intervals for the Pikermi Formation (Ahmatovo Formation). Given the biochronological constraints, the two normal polarity intervals can be identified unambiguously as chrons C3Bn and C3Br.1n of the Neogene geomagnetic polarity timescale ([27]; Fig 8). In detail we assign chron C3Bn to the normal polarity zones of sub-sections Chom A, Chom B, and to the top parts of PV1 and Azmaka. Furthermore, we correlate the lower normal polarity parts of Azmaka and PV1 with chron C3Br.1n and the reversed polarity of the PV3 section with the underlying chron C3Br.2r (Fig 8). The top of Chom B and the thin upper part of the Rafina Formation correspond to the long chron C3Ar. Similarly, the normal polarity of the Pyrgos *Graecopithecus*-horizon can be correlated to chron C3Bn, but not to chron C3Br.1n, which would contain the older Pikermi fauna of PV1 (Fig 8;[51]). Our bio-magnetostratigraphic results imply sediment ages for the Pikermi Formation of between ~7.4 and ~7.0 Ma (upper part of chron C3Br.2r to the lower part of chron C3Ar) and between 7.28 and 7.2 Ma (chron C3Br.1n to the base of chron C3Bn) for the Ahmatovo Formation in Azmaka.

To further refine our age estimations of the Pikermi Formation we apply cyclostratigraphy and astrochronology. We achieve a consistent orbital calibration by tuning the debris flows (Red Conglomeratic Member) and the mid-points of fluvial channel-trains (Chomateri Member) to insolation seasonality maxima (Fig 8). This correlation reveals that patterns of obliquity-precession interference correspond to alternations of thick/dominant and thin/faint debris flows or fluvial channels, respectively (Fig 8), consistent with Mediterranean sapropel cyclicity[52]. Furthermore, the Red Conglomeratic Member corresponds to low-amplitude variation of seasonal insolation contrast between 7.37 and 7.17 Ma (modulated by the long-term 405 kyr/2.4 Ma eccentricity minimum) and the Chomateri Member correlates to high-amplitude insolation seasonality variations (modulated by an eccentricity maximum) between 7.17 and 7.11 Ma. The dominant fluvial channel at the base of the Chomateri Member and the thick gravel directly above the Pyrgos *Graecopithecus*-horizon, therefore, correlates with the first Messinian eastern Mediterranean sapropel at 7.168 Ma (Fig 4A;[53]), which developed during a pronounced seasonal insolation contrast maximum. On the basis of orbital tuning of the Pikermi Formation and the bio-magnetostratigraphy of Azmaka, *Graecopithecus* can be dated to 7.24 Ma (tooth from Azmaka) and 7.18–7.17 Ma (type mandible from Pyrgos) and is, thus, of earliest Messinian age. The levels that contain a classical Pikermi mammal fauna can now be dated to between 7.33 and 7.29 Ma (Fig 8). Therefore, the transition from the Pikermi to post-Pikermi fauna appears to coincide with the Tortonian-Messinian boundary.

### Reconstruction of sedimentary environment

To investigate the depositional environment we analysed grain texture and grain-size distributions of clastic sediments. To further explore potential sources of sediments, we measured salt chemistry and dated detrital zircons.

#### Sediment texture and provenance

**Fig 9:**
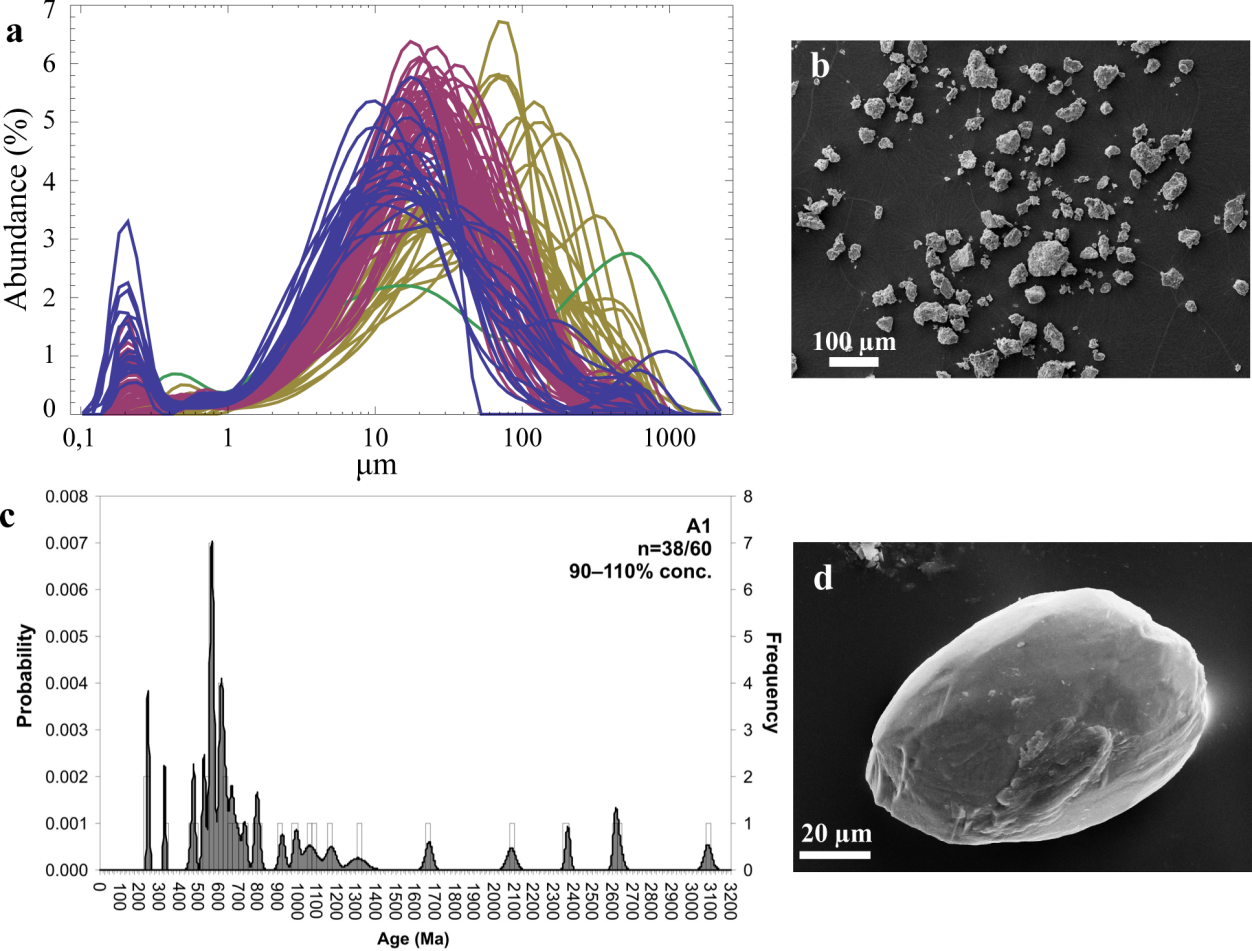
Grain-size and -texture and U-Pb geochronology of detrital zircon grains. **a**, Grain-size distribution (GSD) of siltstone samples (n = 97) of the Pikermi Formation. Each GSD is coloured according to its dominant end-member (blue–EM1, magenta–EM2, yellow–EM3, green–EM4). **b**, SEM image of a siltstone sample (Red Conglomeratic Member, CA 2.75) with angular grains and adhering particles (for texture documentation see Fig 10). **c**, Combined binned frequency and probability density distribution plots of U-Pb LA-ICP-MS ages of detrital zircon grains from sample CA 2.75. **d**, SEM image of a ‘giant’ detrital zircon (sample CA 2.75) with a typical surface shaped by aeolian abrasion.

**Fig 10:**
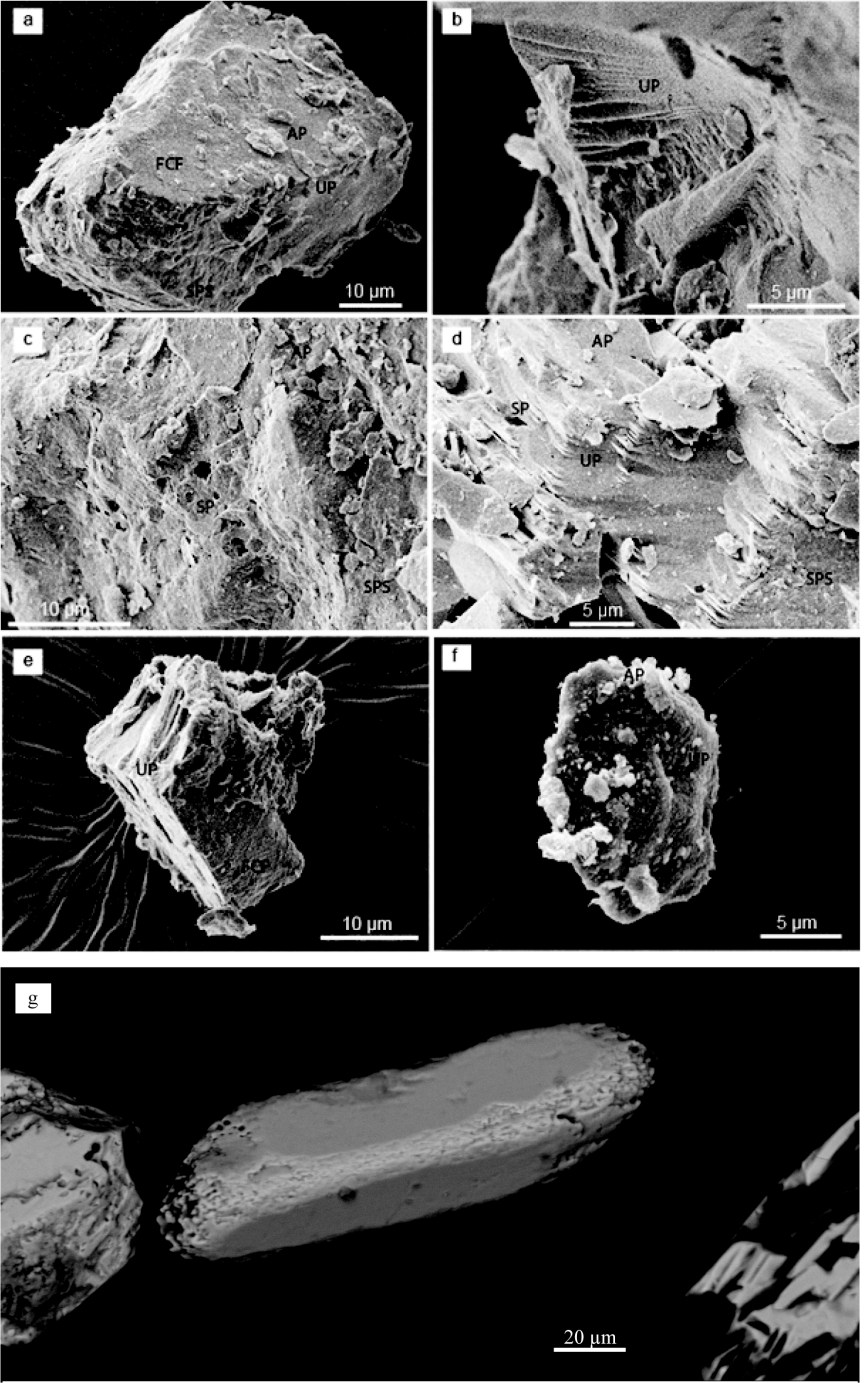
Silt grain texture and aeolian zircons from the studied sediments. **a-f,** Raster Electron Microscope images of silica precipitation surfaces (SPS), solution pits (SP), upturned plates (UP), flat cleavage faces (FCF), and adhering particles (AP). Samples **a**, PV3–0.4, **b**, PV3–0.4, **c**, PV1 0.5, **d**, PV3–0.4, **e**, Pyrgos Vassilissis, and **f**, CA 2.75. **g**, Detrital zircon (CA 2.75 m) affected by chemical corrosion (etching) before transportation.

Silt-sized particles from all samples of the Pikermi Formation (including Pyrgos) are mostly angular to sub-angular and have a high abundance of clays adhering to quartz grains (Fig 9B), whereas some particles are composed solely of adhering clay aggregates, which is typical of loess [18]. Characteristic textures that suggest aeolian transport of silt[18] includes mechanically formed upturned plates, flat cleavage faces and planes, and features of silica solution/precipitation (Fig 10).

**Fig 11:**
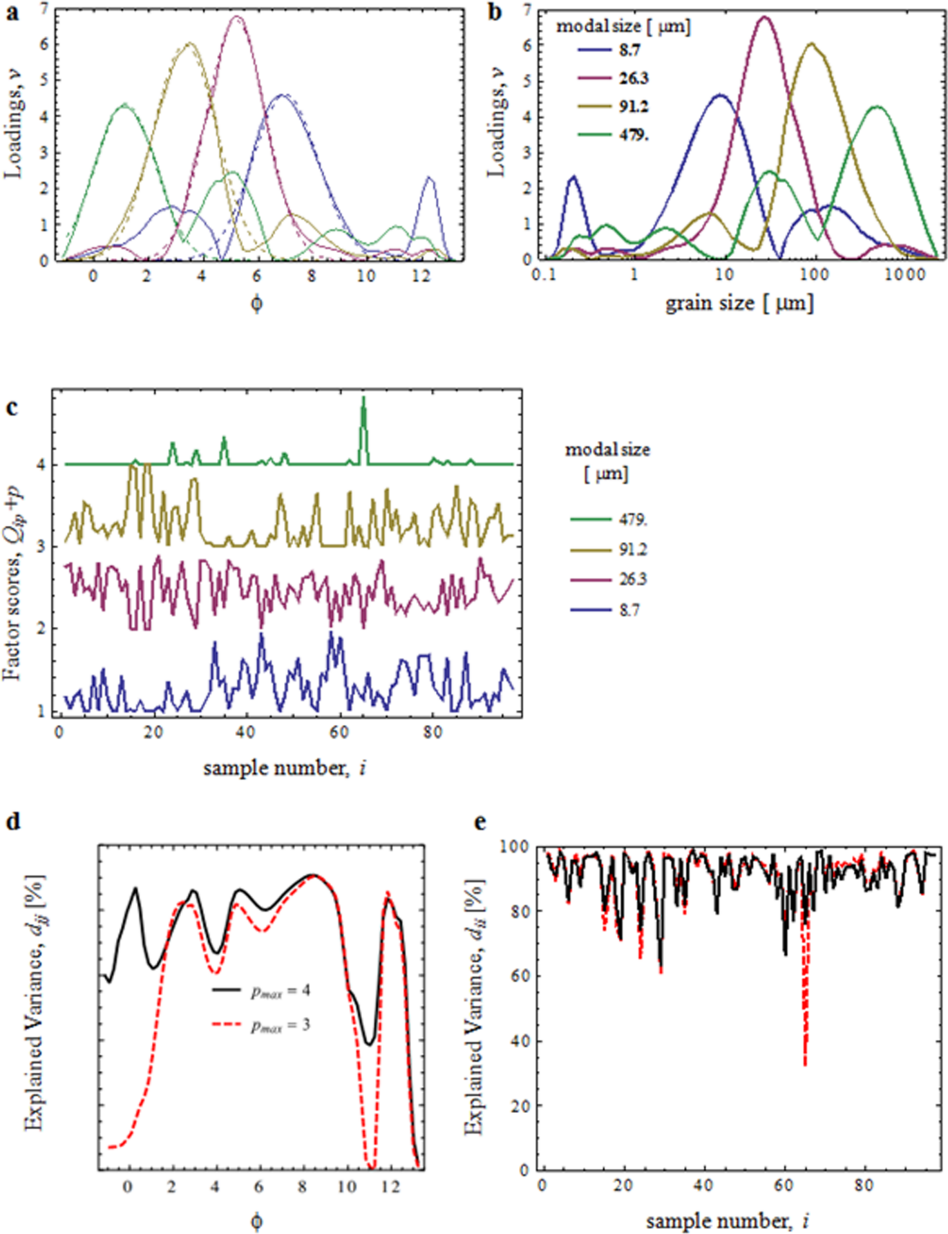
End-members of grain-size distributions from the studied sediments. **a**, Factor loadings (solid lines) of end-members of grain-size distributions obtained using EMMAgeo[57] for 97 samples of the Pikermi Formation (Mesogea Basin). The dashed lines represent Gaussian fits to the main modes and are referred to as simplified EM spectra (summarized in S11 Table). **b**, Same factor loadings, plotted on a micrometer scale. **c**, Relative contributions of EMs to each sample from the Pikermi Formation. For better visibility, the curves are shifted vertically, using the EM index *p* as an offset. **d, e**, Explained variance of the original grain-size data from the Pikermi Formation by the EM model based on four end members (solid lines). **d**, At the level of grain-size (Eq. 4a in S1 Text), **e**, with respect to samples (Eq. 4b in S1 Text). When EM *p* = 4 is omitted from the model (i.e., *p*_max_ = 3, dashed lines), the mean variance at the sample level of 93% is practically not affected, while the mean variance at the grain-size level drops from 72% to 59%, resulting in an overall drop in total explained variance from 83% to 77%.

In SEM images, 40–100 μm detrital zircons are mostly rounded due to aeolian abrasion (Fig 9D). Half of all zircon grains (n = 60) are characterized by traces of etching, which was probably caused by intense chemical weathering before transportation (Fig 10G). Texture analysis results are corroborated by end-member (EM) modelling of grain-size distributions, which reveals a distinct aeolian signature[54, 55] of the Pikermi Formation sediments (Fig 9A). Two aeolian EMs explain 50% of the total variance (Fig 11) and have dominant modes of 9 μm (EM1) and 27 μm (EM2), whereas EM3 and EM4 are in the sand size range and are, therefore, interpreted to be of fluvial origin (Fig 9A). EM1 contains a second distinct mode at 0.2 μm, which does not overlap with any other main mode, and a lesser mode at 100 μm (Fig 11). The same aeolian EMs as in Pyrgos and Pikermi (S2 Fig) are found in most palaeosols of Azmaka and at the top of the Rafina section (S3 and S4 Figs), which demonstrates depositional coherence between the sites. Significantly, a clear aeolian signature is also manifest in grain-size data (S4 Fig) for a 30-m-thick section of red silts from Cucuron (Mt. Luberon) in southern France, which is famous for rich large mammal fauna contemporaneous with Pikermi[56].

#### Ionic composition of TSS

TSS concentrations vary between 0.2% and 4.4%. Na^+^ and Ca^2+^ are the dominant leachable cations in all samples, whereas Cl^-^ and SO_4_^2-^ dominate the anions. Ca^2+^ dominates samples older than 7.24 Ma, whereas Na^+^, Cl^-^ and SO_4_^2-^ dominate in younger Messinian samples, which indicates a change in the source regions of soluble salts. The ratio between anion and cation concentrations (Ʃ^-^/Ʃ^+^) is <1 in all samples and is especially low in Tortonian samples, which confirms the presence of soluble hydrogencarbonate ions (not measurable by ionic chromatography). In all three measured Pyrgos samples anion concentrations are extremely low (Ʃ^-^/Ʃ^+^ = 0.01), bromide is absent, and chloride is present in traces only, which suggest leached conditions.

#### Detrital zircon geochronology

From sample CA 2.75, 60 detrital zircon grains were analysed (S2 Table). In the cathodoluminescence images most zircons have a magmatic oscillatory zoning. Complex zircons are rare. Of these, 38 grains have concordant ages in the range of 90 to 110% (Fig 9, S5 Fig). The youngest concordant grain is 241±7 Ma old. The oldest zircon yields an age of 3,084±38 Ma. Only 18% of all zircon grains are younger than the Precambrian. Two grains are Triassic in age (241±7 and 248±9 Ma), while others have Palaeozoic ages (328±9 to 528±9 Ma). 55% of all zircons in the sample are Neoproterozoic in the age range of ca 560–995 Ma. The probability plot has distinct peaks at ca 560, 615, and 800 Ma (Fig 9, S5 Fig). 11% of all concordant zircons are Mesoproterozoic (ca 1,063–1,307 Ma). Palaeoproterozoic zircon grains make up 8% of all grains and range between ca 1,669 and 2,373 Ma. 8% of all grains are Archaean (ca 2,615 to 3,084 Ma). This age distribution rules out a prominent Laurussian source (Baltica and adjoining terranes)[58–60]. In such areas Mesoproterozoic zircon grains in the age range from 1,000 to 1,600 dominate[61].

#### Source of aeolian sediments

**Fig 12:**
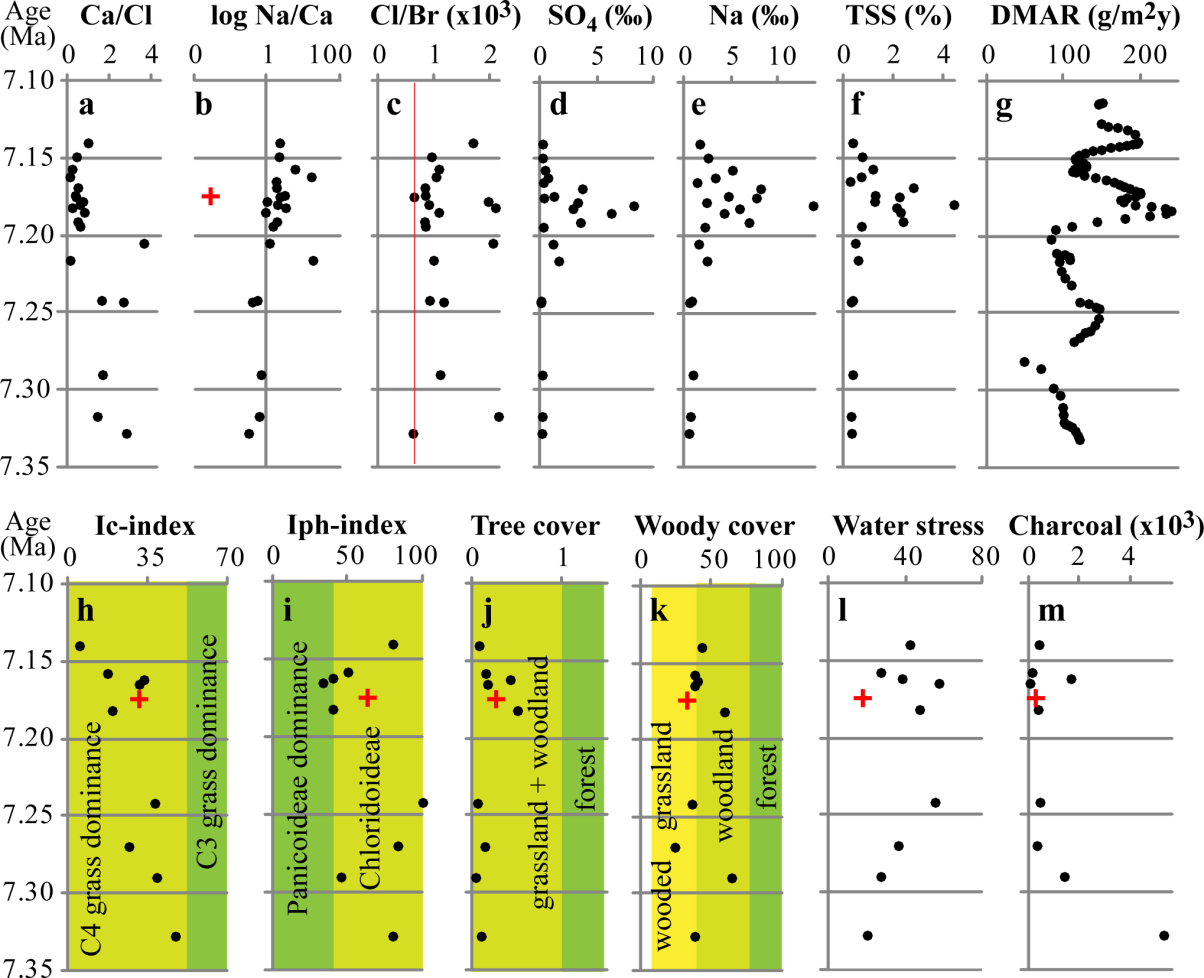
Mineral dust and vegetation of the Pikermi formation and Pyrgos (red crosses). Upper panel–dust mass accumulation rate (DMAR) and total soluble salt (TSS) chemistry. **a**, Ca/Cl and **b**, Na/Ca concentration ratios in the leachate indicate change from Ca^2+^ to Na^+^ and Cl^-^ dominance. Low Na/Ca and high Ca/Cl ratios (140, not shown) for Pyrgos suggest leached conditions. **c**, Cl/Br molar ratios point to contributions from both marine-based and evaporitic sodium chlorides (red line–marine Cl/Br ratio of 655,[23]). Concentrations of (**d**) soluble SO_4_^2-^ and (**e**) Na^+^ in the samples indicate that halite and gypsum dominate TSS during the earliest Messinian. **f**, TSS reaches its highest concentrations at 7.18 Ma. **g**, DMAR is quantified for the proportion of silt <30 μm. Lower panel–phytolith indices and charcoal abundance. **h**, Climatic index (Ic) specifies the relative proportions of C4 and C3 grasses and **i**, the humidity-aridity index (Iph) represents the relative proportion of C4-grass sub-families Panicoideae and Chloridoideae. **j**, Tree cover density index (D/P) is the ratio between woody dicotyledons and grass phytoliths. **k**, Woody cover index describes the relative abundance of globular (woody dicotyledon) phytoliths. **l**, Water-stress index quantifies aridity by the relative percentage of silicified bulliform cells. **m**, Micro-charcoal abundance is given in 10^3^ particles per gram dry-weight.

Field evidence and groundwater chemistry[62] suggest a significant salt content for the Pikermi Formation in the Mesogea Basin (Fig 12, S1 Table). The salinity of the aeolian red silts, measured as TSS concentration, varies between 0.2 and 4.4% (Fig 12F), and is within the range of present-day Saharan dust values of soluble salt (0.1 to 3.1%,[63]). The major ionic components are Cl^-^ and Na^+^, with significant contributions from Ca^2+^ and SO_4_^2-^. Molar ratios (e.g. chloride-bromide and calcium-chloride ratios) record the contribution of both marine-based aerosols and continental salts (Fig 12A–12E), which suggests a dust source region in arid North Africa. This is further corroborated by U-Pb geochronology of aeolian zircons. The majority of concordant zircon grains are Neoproterozoic in age with distinct peaks at 560, 615, and 800 Ma (Fig 9C). Such a zircon population is typical of crustal units that originated during Cadomian, Avalonian, and Pan-African orogenic events[58] and typically occur in Northern Africa[64] and in European Gondwanan fragments. Soluble salt systematics and Gondwana provenance of zircons are consistent with arid North Africa (>600 km further south) as a source of the Pikermian aeolian silt.

#### Dust accumulation

At 7.19 Ma, the DMAR for the silt fraction < 30 μm (Fig 12G) increases from low- (50–150 g/m^2^y) to high-amplitude variations (100–250 g/m^2^y), which is comparable with Pleistocene DMAR values in peridesert loess deposits[65]. The increased DMAR was accompanied by a drastic change in TSS content and chemistry of aeolian silt. During the Tortonian, TSS was low and was dominated by Ca^2+^ (Fig 12A and 12B), whereas earliest Messinian dust (especially during two maxima at 7.18 and 7.157 Ma) was rich in soluble salts (TSS up to 4.4%) dominated by evaporitic minerals (halite, gypsum).

### Reconstruction of biotic environment

#### Phytoliths, palynology and micro-charcoal

**Fig 13:**
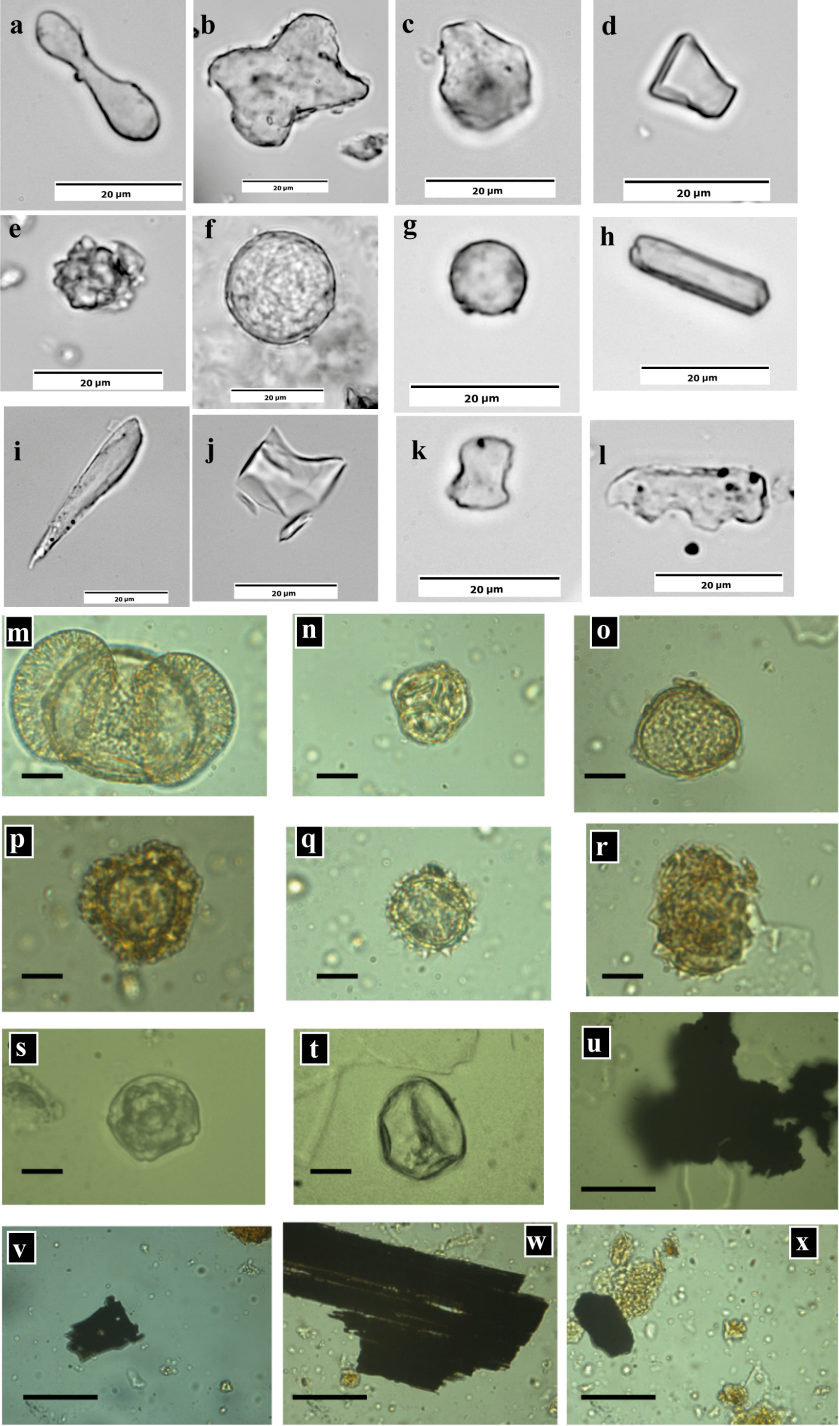
Phytoliths, pollen and micro-charcoal particles. **a-l**, Phytolith types used in this study. **a**, Bilobate, **b**, cross, **c**, fan-shape, **d**, trapeziform short cell, **e**, globular echinate, **f**, globular granulate, **g**, globular psilate, **h**, parallelepiped elongate, **i**, point-shape/acicular, **j**, rondel, **k**, saddle, **l**, trapeziform polylobate. **m-t**, Pollen from sample CA 2.75 (Pikermi Formation; black bar represents 10 μm). **m**, *Pinus*, **n**, Ericipites, **o**, Chenopodiaceae, **p**, Caryophyllacae, **q**, Asteraceae, **r**, Malvaceae, **s**, Alnipollenites, **t**, Poaceae. **u-x** Charcoal particles from sample CA 2.75 in the size-range between 30 and 150 μm (black bar represents 30 μm).

We obtained a rich (n >1.600) and morphologically diverse (>150 morphotypes, ~50 per sample) phytolith record from the Pikermi Formation (Fig 13A–13L; S3 Table), belonging to both C3 and C4 Poaceae (grasses), herbs, woody eudicotylodons, and palms (see S3 Text for details on phytolith taxonomy and interpretation). The phytolith abundance of investigated Azmaka samples was too low (n = 27) for further interpretation. In contrast to phytoliths, pollen is rarely preserved in the Pikermi Formation (Fig 13M–13T; S4 Table). The ten samples investigated contain only 285 pollen grains, which dominantly belong to *Pinus* (50%). The overall occurrence of bisaccate *Pinus* in these aeolian sediments can be attributed to its highly effective dispersal by wind and to its high production rates. *Pinus* is captured by long distance transportation, so the source of *Pinus* pollen cannot be derived from its low numbers in most samples. Pollen types that are more bound to its source have been found in significant numbers only in sample CA 2.75, which has important contributions of Chenopodiaceae (17%), Asteraceae (6%), Poaceae (6%), and *Quercus* (3%). A similar pollen composition has been found by ref. [34] from the Rafina Formation. Sediment from the *Graecopithecus*-bearing level of Pyrgos produced only five pollen grains, belonging to *Pinus*, Chenopodiaceae, Poaceae and *Ulmus*/*Zelkova*.

Charcoal preserves well (Fig 13U–13X; S4 Table) in the Pikermi Formation and, therefore, can be used as a record of past fires[66]. CP were present in all samples. Most CP are smaller than 30 μm, often with frazzled margins. Only some of the bigger CP have wood structures (Fig 13W), which argues against the presence of forest ecosystems within the storage period. The CP concentration is highest in the oldest sample (PV3-0.60) of the sequence (>5000/gram), and declines rapidly to generally lower values during the Messinian, which suggests a decreasing fire frequency.

#### Stable isotopes of pedogenic carbonates

Pedogenic carbon isotope data (S5 Table) range between -3.03 ‰ and -4.38 ‰, which is consistent with high C4-biomass (according to mass balance proposed by ref. [67], 52–43% C4-biomass) and are interpreted to represent wooded grassland[68] with low woody canopy cover[69].

#### Pikermian and post-Pikermian vegetation and ecosystems

To obtain deeper insight into the *Graecopithecus* environment we reconstructed the vegetation of the Pikermi Formation (including Pyrgos) based on the rich and morphologically diverse phytolith record (Fig 13). The grass communities were continuously dominated by C4-grasses and the proportions of C3-grass decreased during the Messinian (Fig 12H). Within C4-grasses, Chloridoideae (short grass) prevailed, except during times of highest dust accumulation, when Panicoideae (long grass) became more common (Fig 12I). Phytolith indices (Fig 12J and 12K) strongly suggest open, grass-dominated habitats with woody cover estimates[70] of 40 ±12%, which is confirmed by rarely preserved pollen grains of Chenopodiaceae and Asteraceae. Phytolith based C4 biomass estimations (S3 Table) range between ~30% during the latest Tortonian to over 50% in the earliest Messinian in accordance with palaeosol δ^13^C data (S5 Table). These results imply a savannah biome for the Pikermi Formation that ranged around the wooded grassland to woodland transition[71]. Furthermore, increased silicified bulliform cell abundance from the latest Tortonian toward the earliest Messinian (Fig 12L) documents progressively prolonged water-stress durations, related to increased climatic water deficit[72]. Parallel to this trend the abundance of microcharcoal particles, and thus wild-fire frequency, decreased (Fig 12M), which may suggest that fuel availability rather than climate drove fire activity, similar to present-day Mediterranean ecosystems[73]. The phytolith record of the *Graecopithecus*-horizon in Pyrgos is in accord with the general picture (red crosses in Fig 12), except for the low water-stress value, which may be related to locally elevated soil moisture directly above the gently northwest dipping Attica detachment fault that likely guided the fluvial drainage of the Athens Basin (Figs 1 and 2).

## Conclusion

For the first time we demonstrate that C4 grasses were the dominant herbaceous element of the Pikermi Formation. Our habitat reconstruction suggests fire-prone woody grasslands and woodlands within a savannah biome for Pikermi and Pyrgos and, thus, provides unambiguous evidence for the early environmental conjectures of Gaudry[36]. Given the potential hominin nature of *Graecopithecus freybergi*, our habitat reconstruction for the Pikermi Formation further supports the “Savannah Hypothesis” put forward to explain earliest hominin emergence[69, 71, 74]. Analysis of both potential hominin sites indicates that *Graecopithecus* inhabited different habitats, be it open braided-river landscapes in Azmaka[4], or the wooded grassland of Pyrgos.

The Tortonian-Messinian transition in the Mediterranean appears to represent a period of significant environmental and climatic changes. During the latest Tortonian (~7.4–7.25 Ma) C4 grass ecosystems progressively penetrate the Balkan Peninsula and constitute the environment of the mammal fauna of Pikermi, which contradicts earlier assumptions [75, 76]. The classical Pikermi fauna is terminated at the beginning of the Messinian (7.25–7.10 Ma) by a significant faunal turnover (post-Pikermi turnover), accompanied by massive increase of Saharan dust and salt accumulation with profound effects on soil salinity and nutrition.

Our results reveal formerly unrecognized Mediterranean environmental changes during the Tortonian-Messinian transition, which provide important constraints for the evolution of *Graecopithecus freybergi*. At the Tortonian-Messinian boundary (7.25 Ma), water-stress levels increased and wildfire frequency decreased, which can be interpreted as increasing aridification. Rather than representing a local phenomenon, aridification occurred on a larger scale. We demonstrate that aeolian dust accumulation was widespread at the northern Mediterranean coast and that large amount of salt-laden mineral dust and marine-based aerosols were blown from dried lake beds in North Africa toward Europe, where ~30-m-thick red silts were deposited in southern Greece and southern France. We relate this dust accumulation to progressive late Tortonian Mediterranean aridification and cooling, which started at around 7.4 Ma and culminated during the earliest Messinian, when Mediterranean Sea surface temperature dropped by about 7°C to values comparable to the present-day (Fig 4,[41, 77, 78]). Modelling studies[79] have shown that Middle Miocene Tethyan seaway closure and accelerated Late Miocene uplift of the Iranian plateau[80] provided key boundary conditions for north African aridity. We hypothesize that the ~700 kyr cooling episode[41], combined with the long-term eccentricity minimum between 7.3 and 7.2 Ma, acted as a final trigger for substantial north African aridization, which resulted in the initial formation of a large Saharan and Arabian desert belt[79, 81]. Furthermore, mineral dust in Attica was rich in soluble evaporites (halite, gypsum) in the earliest Messinian and especially during two pronounced insolation seasonality minima at 7.18 and 7.157 Ma, which suggests an orbitally driven progressive Sahara desertification. We suppose that a latest Tortonian to early Messinian dust- and salt-laden atmosphere over the Mediterranean may have further accelerated cooling and aridification via absorption of incoming solar radiation and, thus, may partially explain regionally accentuated Mediterranean cooling[41].

The documented environmental changes were likely to have caused a significant faunal transition. Our dating of *Graecopithecus* and the taxonomy of its accompanying large mammals indicate that, during culmination of cooling at the base of the Messinian, the post-Pikermi turnover replaced part of the Pikermi fauna. Several newcomers like the elephantoid *Anancus* or the boselaphid *Tragoportax macedoniensis* have Asian affinities and we hypothesize that Eastern Mediterranean aridification played an important role in the westward shift of their habitats. *Graecopithecus*, as part of this new post-Pikermi fauna, lived in a warm-temperate and dusty environment unlike any other known hominid (except for our own genus). *Graecopithecus* predates by several hundred thousand years the next youngest candidate hominin *Sahelanthropus*, which occupied the southern Saharan tropics after its earliest Messinian desertification [82]. Given the potential hominin affinity of *Graecopithecus*, our results suggest that the *Pan*-*Homo* split predated the Messinian and that the chimpanzee–human last common ancestor thrived in the Mediterranean region. The emerging Saharan and Arabian desert belt thereby possibly acted as a vicariant agent[83]. Our conclusions support views[3] that major Miocene hominid radiations occurred outside Africa and endorse the hypothesis[5] that the hominin clade arose in the Eastern Mediterranean.

## Supporting information

S1 Fig Geopedal structures in giraffid long-bones from Pyrgos Vassilissis. Sediment infill of bones (a, TE 124; b, TE 130) overgrown by geopetal sparry calcite, which provides a palaeo-horizon for palaeomagnetic analysis of Pyrgos.
(TIF)

S2 Fig Grain-size spectra for palaeosol samples from the *Graecopithecus* horizon of Pyrgos (Pikermi Formation, Attica Basin). Measured grain size distribution (GSD, shown as a grey area in each diagram) versus fitted grain spectra (red line) using the simplified end-member (EM) spectra (Fig 11) from the Pikermi Formation of the nearby Mesogea basin (EM1—blue, EM2—magenta, EM3—brown, EM4 green). The r^2^ value for each diagram represents the variance of the GSD explained by the end members.
(EPS)

S3 Fig Grain-size spectra for samples from Azmaka palaeosoils (Ahmatovo Formation, Upper Thrace Basin). Measured GSD versus fitted grain spectra using simplified end-member (EM) spectra from the Pikermi Formation (Mesogea Basin) for the aeolian components only (EM1—blue, EM2—magenta). The r2 value for each diagram represents the variance of the GSD explained by the two EMs. The aeolian contribution is documented for Azmaka samples 6b (d), 8 (e; *Graecopithecus* horizon), and 12a (g), corresponding to profile meters 14, 16.5, and 24.5 in Fig 8.
(EPS)

S4 Fig Grain-size spectra for samples from Rafina (top of Rafina Formation, Mesogea Basin) and Cucuron (Mt Luberon, Vaucluse, France). Measured GSD versus fitted grain spectra using the simplified EM spectra from the Pikermi Formation (Mesogea Basin) for aeolian components only (EM1—blue, EM2—magenta). The r2 value for each diagram represents the variance of the GSD explained by these two end members. The aeolian contribution is documented for both samples.
(EPS)

S5 Fig Ages and age distribution of aeolian zircons from the Pikermi Formation (Mesogea Basin). a, b, Concordia plots of U-Pb LA-ICP-MS data for sample CA 2.75 for all measurements (a) and of the younger zircon grains in an age range of 0 to 1500 Ma (b). c, d, Combined binned frequency and probability density distribution plots of U-Pb LA-ICP-MS ages of detrital zircon grains from sample CA 2.75. Data are shown in the ranges of 0 to 3200 Ma (c) and 0 to 1500 Ma (d).
(TIF)

S1 Table Ionic composition of soluble salts from eolian silt of the Pikermi Formation (Attica and Mesgea Basins). (XLSX)

S2 Table LA-SF-ICP-MS U-Pb-Th data of detrital zircons. Sample CA 2.75, n = 60 measured zircon grains, red siltstone, Pikermi Formation, Red Conglomeratic Member, sub-section Chomateri A (coordinates: N 38° 00’ 48”, E 23° 57’ 46”). Grey shading indicate recommended ages in the range of concordance of 90–110%. Discordant ages (below or above the range of concordance of 90–110%) are indicated by italic font style.
(XLSX)

S3 Table Detailed counts of phytolith types (in %) and phytolith indices from the Pikermi Fm (including Pyrgos), Attica and Mesgea Basins. (XLSX)

S4 Table Detailed counts of pollen and micro-charcoal from the Pikermi Formation (Athens and Mesogea Basins). (XLSX)

S5 Table Pedogenic carbonate δ^18^O and δ^13^C data from the Pikermi Formation (Athens and Mesogea Basins). (XLSX)

S6 Table Measurements of *Hippotherium brachypus* from Pyrgos Vassilissis. Dimensions (in mm) of the cranial fragment AMPG 02, the premolar row TE 114, and the mandibular fragment AMPG 03. The definitions of measurements (italics) follows [84].
(XLSX)

S7 Table Measurements of *Hippotherium brachypus* from Pyrgos Vassilissis. Dimensions (in mm) of the upper and lower check teeth. The measurements L (length) and W (width) are taken on the occlusal surface and follows [84].
(XLSX)

S8 Table Giraffid mandibular tooth dimensions. cf. *Palaeotragus* sp. from Pyrgos Vassilissis, compared to *Palaeotragus rouenii* and *Bohlinia attica* (MNHN: Muséum National d’Histoire Naturelle, Paris) (in mm; L–length, W–width, H—height). Heights of unworn teeth are in bold.
(XLSX)

S9 Table Measurements of *Tragoportax macedoniensis* from Pyrgos Vassilissis and Dytiko (DTK, DIT, DKO). Horn-core basal dimensions (in mm). APD–anteroposterior diameter of the horn-core at its base; TD–transverse diameter of the horn-core at its base. Values in parentheses indicate slightly approximate measurements. Data from Dytiko according to [85].
(XLSX)

S10 Table Measurements of *Tragoportax macedoniensis* from Pyrgos Vassilissis and Dytiko (DTK, DIT, DKO). Dimensions (in mm, L–length) of the upper tooth series. Values in parentheses indicate approximate measurements. Data from Dytiko according to [85].
(XLSX)

S11 Table Sedimentological and statistical properties of end-member loadings obtained with EMMAgeo. (DOCX)

S1 Text End member modelling of grain-size spectra. (DOCX)

S2 Text U-Th-Pb isotopes. (DOCX)

S3 Text Phytolith taxonomy and interpretation. (DOCX)

S4 Text Sedimentology. (DOCX)

S5 Text The Pyrgos Vassilissis mammalian fauna. (DOCX)
