# Supplementary figures and images for: Messinian age and savannah environment of the possible hominin Graecopithecus from Europe
Source: PLoS One. 2017 May 22;12(5):e0177347. doi: 10.1371/journal.pone.0177347 (PMC5439672; doi:10.1371/journal.pone.0177347)

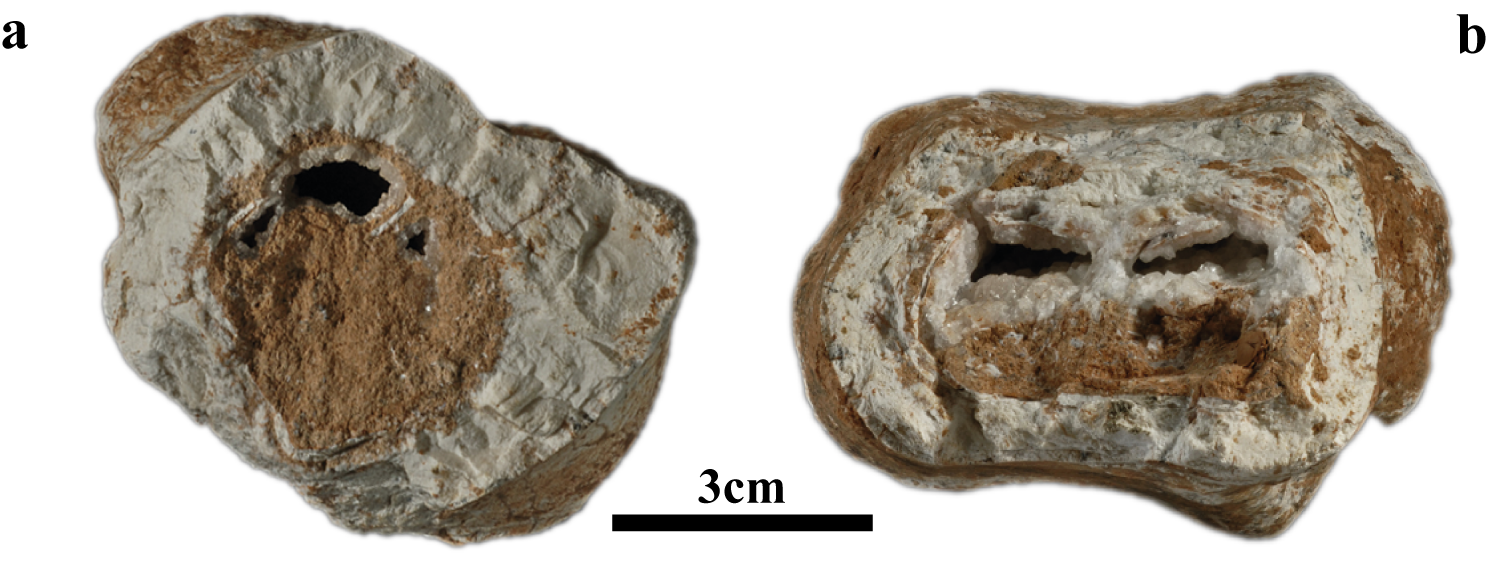

Supplement: S1 Fig — Sediment infill of bones (a, TE 124; b, TE 130) overgrown by geopetal sparry calcite, which provides a palaeo-horizon for palaeomagnetic analysis of Pyrgos. (TIF) [file pone.0177347.s001.tif]

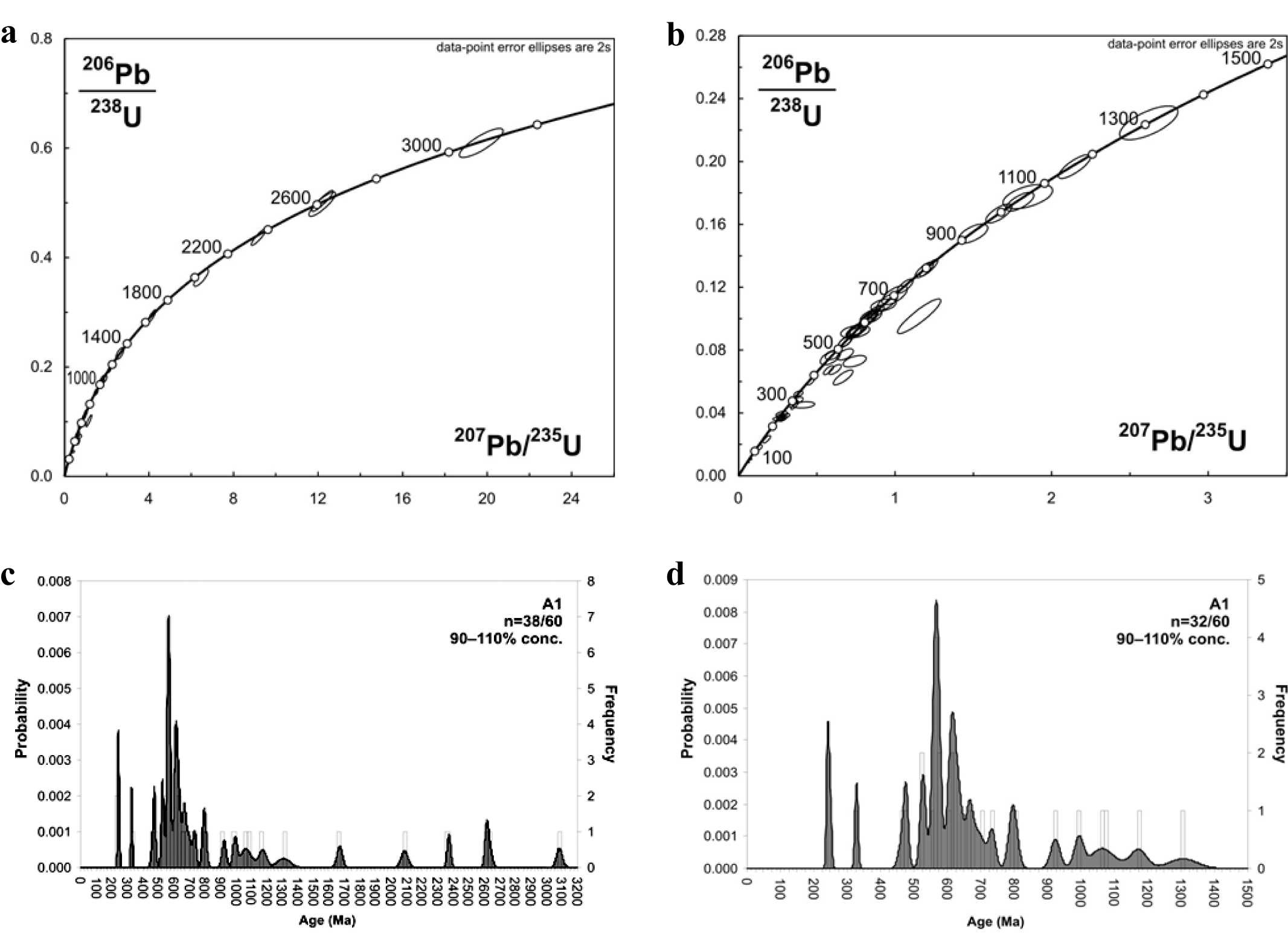

Supplement: S5 Fig — a, b, Concordia plots of U-Pb LA-ICP-MS data for sample CA 2.75 for all measurements (a) and of the younger zircon grains in an age range of 0 to 1500 Ma (b). c, d, Combined binned frequency and probability density distribution plots of U-Pb LA-ICP-MS ages of detrital zircon grains from sample CA 2.75. Data are shown in the ranges of 0 to 3200 Ma (c) and 0 to 1500 Ma (d). (TIF) [file pone.0177347.s005.tif]
