# Supplementary material for: Messinian age and savannah environment of the possible hominin Graecopithecus from Europe
Source: PLoS One. 2017 May 22;12(5):e0177347. doi: 10.1371/journal.pone.0177347 (PMC5439672; doi:10.1371/journal.pone.0177347)
Supplement: S11 Table — (DOCX) [file pone.0177347.s016.docx]

**S11 Table. Sedimentological and statistical properties of end-member loadings obtained with EMMAgeo.**

| EM *p* | Particle size class | Modal Size | Dominant modes (Gaussian fit in φ space) | | | Var(Q_p_)  [% of Total  Expl. Var] |
| --- | --- | --- | --- | --- | --- | --- |
|  |  | [μm] | <x>[μm] | <x> [φ] | σ_x_ [φ] |  |
| 1 | fine/medium silt | 8.7 | 7.87 | 6.99 | 1.20 | 30.9 |
|  | colloid | 0.21 | 0.21 | 12.2 | 0.35 |  |
| 2 | coarse silt | 26.3 | 27.44 | 5.19 | 1.08 | 28.9 |
| 3 | very fine sand | 91.2 | 99.54 | 3.33 | 1.16 | 35.6 |
| 4 | coarse sand | 479 | 451.3 | 1.15 | 1.11 | 4.6 |
