# Supplementary material for: Messinian age and savannah environment of the possible hominin Graecopithecus from Europe
Source: PLoS One. 2017 May 22;12(5):e0177347. doi: 10.1371/journal.pone.0177347 (PMC5439672; doi:10.1371/journal.pone.0177347)
Supplement: S1 Text — (DOCX) [file pone.0177347.s017.docx]

**End Member Modelling of grain-size spectra**

End-member analysis was performed to identify sedimentologically distinct components (end members) in the grain-size data set and study the temporal variations of the component fractions. The underlying assumption is that the variations in the measured grain-size distribution along the sampled profiles largely represent variations in the physical mixing ratios of a few end-member grain-size distributions so that the original data set $y_{\mathrm{ij}}$ (row index i=1,…, m samples and column index j=1,..., n grain size bins) can be approximated by a linear mixing model,

or, in index notation,

where $p_{\max}\ll m$ is the number of end-members, $Q_{\mathrm{pi}}$ is the relative contribution of the p-th end member to i-th sample, ${v_{p}}^{T}=(v_{p1,}\ldots,v_{pn,})$is the grain-size distribution of the p-th end member, which is defined on the same discrete grain-size bins $\phi_{j=1,\ldots,n}$ as the data set, and $\tilde{\epsilon}=\epsilon_{\mathrm{ij}}$ is the matrix of the unexplained residuals. Since end-member analysis has its roots in factor analysis, it is customary to refer to the component fractions $Q_{\mathrm{pi}}$ as factor scores and to the $v_{\mathrm{pj}}$as factor loadings. We refer to the expression

as predicted data. The following normalizations apply to Eq. (1):

Here we sketch the two essential steps of procedure for obtaining the $v_{p}$ vectors: First, the full set of eigenvectors up (p=1,…,m) of the outer product matrix ${{y_{\mathrm{ij}}}^{T}y}_{\mathrm{ij}}$ is reduced to those with significant eigenvalues (Note: The eigenvector with the largest eigenvalue is just the mean grain-size spectrum over all samples). The $v_{p}$ are obtained in the next step by rotating the reduced set of $v_{p}$ so as to maximize the varimax criterion defined by Kaiser (1958). Details of the procedure, including quantile transformations of the data, are described in ^1^. The factor scores Qpi in Eq. (1) represent regression weights and are obtained by minimizing the residuals between original data and predicted data,

under the constraint that $Q_{\mathrm{ip}}\geq0$. The total correlation coefficient between observed data and predicted data is given by


where $r_{\phi}^{2}$ is the variable-wise correlation coefficient

with $d_{jj}$ representing the diagonal matrix elements of the correlation matrix between the columns of matrix $\tilde{y}_{ij}$ and $y_{ij}$, and $r_{s}^{2}$ is the sample-wise correlation coefficient

We used the R-package EMMAgeo ^2^ to obtain an estimate of robust end member (EM) factor loadings *v_p_*. To obtain an independent estimate, we also performed end-member analysis using self-written code in Wolfram-Mathematica. Either way, we find that *p*_max_ = 4 end members representing five different modes (because of bimodality of one end member, see Figs 11d and 11e) are sufficient to obtain a total correlation coefficient *r^2^* between observed and predicted data of between 80% and 85%, depending on the details of the least-squares fitting.

For each EM, the factor loadings have a predominant mode of Gaussian shape in φ space, corresponding to a lognormal shape in μ space (Figs 11a and 11b). Smaller side maxima overlap with the main modes of the factor loadings of other EMs. The only exception here is EM1 with main mode at about 7 φ (7.9 μm) and a second distinct mode at 12.2 φ (0.2 μm) which does not overlap with any other main mode. To characterize the dominant modes, we fitted a Gaussian distribution (with φ as variable) to each major mode contained in the factor loadings (dashed lines in Fig 11a). The results are summarized in S11 Table. It can be seen from S11 Table that EM4 accounts for less than 5% of the total variance in the linear mixing model. Even so, the punctuated presence of this coarse-grain sand component in the record (green line in Figs 11a-c) is an important proxy of fluvial deposition and hence wet conditions in the basin.

**References**

1 Dietze, E. *et al.* An end-member algorithm for deciphering modern detrital processes from lake sediments of Lake Donggi Cona, NE Tibetan Plateau, China. *Sedimentary Geology* **243–244**, 169-180, doi:<http://dx.doi.org/10.1016/j.sedgeo.2011.09.014> (2012).

2 Dietze, M. & Dietze, E. in *EGU General Assembly Conference Abstracts* Vol. 15 2779 (2013).
