# Supplementary material for: Messinian age and savannah environment of the possible hominin Graecopithecus from Europe
Source: PLoS One. 2017 May 22;12(5):e0177347. doi: 10.1371/journal.pone.0177347 (PMC5439672; doi:10.1371/journal.pone.0177347)
Supplement: S2 Text — (DOCX) [file pone.0177347.s018.docx]

**U-Th-Pb isotopes**

Each LA-SF ICP-MS analysis consisted of approximately 15 s background acquisition followed by 30 s data acquisition, using a laser spot-size of 25 and 35 µm, respectively. A common-Pb correction based on the interference- and background-corrected ^204^Pb signal and a model Pb composition ^1^ was carried out if necessary. The necessity of the correction is judged on whether the corrected ^207^Pb/^206^Pb lies outside of the internal errors of the measured ratios. Discordant analyses were generally interpreted with care. Raw data were corrected for background signal, common Pb, laser induced elemental fractionation, instrumental mass discrimination, and time-dependant elemental fractionation of Pb/Th and Pb/U using an Excel® spreadsheet program developed by Axel Gerdes (Institute of Geosciences, Johann Wolfgang Goethe-University Frankfurt, Frankfurt am Main, Germany). Reported uncertainties were propagated by quadratic addition of the external reproducibility obtained from the standard zircon GJ-1 (~0.6% and 0.5-1% for the ^207^Pb/^206^Pb and ^206^Pb/^238^U, respectively) during individual analytical sessions and the within-run precision of each analysis. Concordia diagrams (2σ error ellipses) and concordia ages (95% confidence level) were produced using Isoplot/Ex 2.49 (Ludwig, 2001) and frequency and relative probability plots using AgeDisplay ^2^. The ^207^Pb/^206^Pb age was taken for interpretation for all zircons >1.0 Ga, and the ^206^Pb/^238^U ages for younger grains. For further details on analytical protocol and data processing see ^3^. Th/U ratios are obtained from the LA-ICP-MS measurements of investigated zircon grains. U and Pb content and Th/U ratio were calculated relative to the GJ-1 zircon standard and are accurate to approximately 10%. Analytical results of U-Th-Pb isotopes and calculated U-Pb ages are given in Supplementary file 4. SEM and Cathodoluminescence images (CL) were performed with an EVO 50 scanning electron microscope (Zeiss) at Senckenberg Naturhistorische Sammlungen Dresden.

**References**

1 Stacey, J. S. & Kramers, J. D. Approximation of terrestrial lead isotope evolution by a two-stage model. *Earth and planetary science letters* **26**, 207-221 (1975).

2 Sircombe, K. N. AgeDisplay: an EXCEL workbook to evaluate and display univariate geochronological data using binned frequency histograms and probability density distributions. *Computers & Geosciences* **30**, 21-31, doi:<http://dx.doi.org/10.1016/j.cageo.2003.09.006> (2004).

3 Gerdes, A. & Zeh, A. Combined U–Pb and Hf isotope LA-(MC-)ICP-MS analyses of detrital zircons: Comparison with SHRIMP and new constraints for the provenance and age of an Armorican metasediment in Central Germany. *Earth and Planetary Science Letters* **249**, 47-61, doi:<http://dx.doi.org/10.1016/j.epsl.2006.06.039> (2006).
