# Supplementary material for: Messinian age and savannah environment of the possible hominin Graecopithecus from Europe
Source: PLoS One. 2017 May 22;12(5):e0177347. doi: 10.1371/journal.pone.0177347 (PMC5439672; doi:10.1371/journal.pone.0177347)
Supplement: S3 Text — (DOCX) [file pone.0177347.s019.docx]

**Phytolith taxonomy and interpretation**

*Grass phytoliths (GSSC): Panicoideae and Chloridoideae (C4 Poaceae).* Following ^1-4^ we identify Panicoideae based on two phytolith morphotypes: cross [ICPN], and bilobate short cell [ICPN] (=dumbbell), whereas Chloridoideae will be identified by the saddle [ICPN] phytolith morphotype. The bilobate morphotype is generally characteristic for Panicoideae ^1,5^, but also occur in Chloridoideae ^2,6,7^, as well as some Arundinoideae (*Aristida*, C4), Pooideae (*Stipa*, C3), and some C3 Bambusoideae ^8^. Detailed bilobate morphotype classification is given by ^7,9^.

*Pooideae (C3 Poaceae).* According to ^1,2,10^ pooid grasses will be identified by the rondel [ICPN], trapezoid short cell [ICPN] and trapeziform polylobate [ICPN] phytolith morphotypes. Barboni and Bremond ^11^ report that rondel-types with a base diameter of >15µm can be found only in C3-Pooideae, whereas rondel-types with base diameters <15µm occur in several grass subfamilies, including the Pooideae. The same authors further report that trapezoid short cell and polylobates may also occur in other grass subfamilies (see also ^9^).

*Additional grass indicators.* Grass phytoliths not diagnostic to lower level taxonomy occur also in root cells as well as epidermal cell ^12^. We will use the following morphotypes as (unspecified) grass indicators: Acicular [ICPN] point-shaped and bulliform fan-shaped [gepeg] (epidermal grass phytoliths from bulliform cells according to ^1,4^.

*Palm phytoliths.* Arecaceae are generally identified by the spherical crenate phytolith morphotype (= spiky star ^13^; = spheroid/globular echinate; ^4,12,14-16^.

*Wood phytoliths.* Non-grass phytoliths from woody eudicotylodons are difficult to characterize to taxonomic lower levels, because of their high morphologic diversity within and between families or genera ^12,13^. To identify woody cover in phytolith samples several approaches have been proposed, not all of them are applicable to Neogene Eurasian (Eastern Mediterranean) phytolith associations. In general, most workers accept spheroid/globular shaped phytoliths as forest indicators ^17^. Barboni et al. ^10^ found that the sum of globular echinate (palms), globular granulate, and globular smooth corresponds best with satellite tree cover data in tropical Africa (see globular-index below). In fully awareness of the problems to identify wood-indicating phytoliths in the Neogene of the Eastern Mediterranean, in our study we use the globular (granulate, smooth) morphotypes to characterise eudicotylodon woody cover.

*Phytolith assemblage interpretation:* To quantitatively interpret vegetation change from phytolith assemblages, several indices have been introduced and successfully applied.

*Tree cover density index (D/P).* The D/P index is the ratio of the spherical rough phytoliths, produced by tropical woody dicotyledons, to the sum of Poaceae phytoliths (bilobate short cell, cross, saddle, point-shaped and fan-shaped types). It was first used by ^18^ in West and Central Africa and was later successfully applied to other African lowland ecosystems ^10,19^. High D/P values between 4 and 7 are characteristic for densely vegetated evergreen and semi-evergreen forests ^10,18^, whereas low values (<1) characterize woodlands and grasslands.

*Humidity-aridity index (Iph).* This index was introduced by ^20^ and exemplifies the relative proportion of C4-grasses [Iph: (chloridoid/(chloridoid+panicoid))% ]. This index is widely used to analyse vegetation changes (C4-short grasses versus C4-tall grasses) in North America and Africa ^18,19,21-23^. High Iph values (> 20–40%) indicate Chloridoideae dominated xeric grasslands, whereas low Iph values (< 20–40%) indicate higher percentage of Panicoideae in mesic grasslands ^22^. However, Bremond et al. ^23^ found that Iph may not be a relevant indicator of Chloridoideae versus Panicoideae when (as in mountainous African vegetation) Arundinoideae, Bambusoideae, and Pooideae grasses are present.

*Water-stress index (Fs).* This index was introduced by ^22^ and indicates the relative proportion of (Fs - Fan-shaped; = bulliform) epidermal grass phytoliths from bulliform cells [Fs: (bulliform/(bulliform+GSSC-elongate))% ]. The Fs index was found to be positively correlated with moisture stress (measured as AET/PET, i.e. the ratio of annual actual evapotranspiration to annual potential evapotranspiration) in non-wetland environments and is commonly regarded as indicator for transpiration rates in grass epidermal cells ^22,24-26^.

*Climatic index (Ic).* This index measure the relative proportions of C3 grasses (Pooidae) and was introduced in the North American Great Plains ^5,27^. It represents the ratio of pooid versus all GSSC phytoliths [Ic: (rondel+trapeziform short cell+polylobate/GSSC)%]. High Ic values indicate a dominance of Pooidae within a cooler climate.

*Globular index*. An index introduced by ^10^ to trace tree cover density. The index represent sum of globular morphotypes (echinate, granulate, smooth) relative to all grass (except elongate) phytolith types ([ICPN] nomenclature: bilobate, cross, saddle, rondel, trapeziform polylobate, trapeziform short cell, bulliform fan shape [gepeg], and acicular). In African lowland vegetation the abundance of globular phytoliths almost reproduces the percent tree cover based on satellite data ^10^.

*C4-biomass:* Rough estimations of C4-biomass (S3 Table) we perform using results for relative proportion of C3-grasses (Ic-index) and tree cover density (globular-index; which correlate well with satellite estimates of the tree cover ^10^) according to the following equation: %C4 = (1-Ic) x (1-globular).

**References**

1 Twiss, P. C., Suess, E. & Smith, R. M. Morphological Classification of Grass Phytoliths1. *Soil Science Society of America Journal* **33**, 109-115, doi:10.2136/sssaj1969.03615995003300010030x (1969).

2 Mulholland, S. C. Phytolith shape frequencies in North Dakota grasses: a comparison to general patterns. *Journal of Archaeological Science* **16**, 489-511, doi:<http://dx.doi.org/10.1016/0305-4403(89)90070-8> (1989).

3 Fredlund, G. G. & Tieszen, L. T. Modern Phytolith Assemblages from the North American Great Plains. *Journal of Biogeography* **21**, 321-335, doi:10.2307/2845533 (1994).

4 Kondo, R., Childs, C. & Atkinson, I. *Opal phytoliths of New Zealand*. Vol. 85 (Manaaki Whenua Press: Lincoln, NZ, 1994).

5 Twiss, P. C. in *Phytolith Systematics: Emerging Issues* (eds George Rapp & Susan C. Mulholland) 113-128 (Springer US, 1992).

6 Brown, D. A. Prospects and limits of a phytolith key for grasses in the central United States. *Journal of Archaeological Science* **11**, 345-368, doi:<http://dx.doi.org/10.1016/0305-4403(84)90016-5> (1984).

7 Lu, H. & Liu, K.-B. Morphological variations of lobate phytoliths from grasses in China and the south-eastern United States. *Diversity and Distributions* **9**, 73-87, doi:10.1046/j.1472-4642.2003.00166.x (2003).

8 Piperno, D. R. & Pearsall, D. M. *The silica bodies of tropical American grasses: morphology, taxonomy, and implications for grass systematics and fossil phytolith identification*. (Smithsonian institution press Washington, DC, 1998).

9 Fahmy, A. G. Diversity of lobate phytoliths in grass leaves from the Sahel region, West Tropical Africa: Tribe Paniceae. *Plant Systematics and Evolution* **270**, 1-23, doi:10.1007/s00606-007-0597-z (2008).

10 Barboni, D., Bremond, L. & Bonnefille, R. Comparative study of modern phytolith assemblages from inter-tropical Africa. *Palaeogeography, Palaeoclimatology, Palaeoecology* **246**, 454-470, doi:<http://dx.doi.org/10.1016/j.palaeo.2006.10.012> (2007).

11 Barboni, D. & Bremond, L. Phytoliths of East African grasses: An assessment of their environmental and taxonomic significance based on floristic data. *Review of Palaeobotany and Palynology* **158**, 29-41, doi:<http://dx.doi.org/10.1016/j.revpalbo.2009.07.002> (2009).

12 Piperno, D. R. *Phytoliths: a comprehensive guide for archaeologists and paleoecologists*. (Rowman Altamira, 2006).

13 Kondo, R. Opal phytoliths, inorganic, biogenic particles in plants and soils. *Japan Agricultural Research Quarterly* **11**, 198-203 (1977).

14 Runge, F. The opal phytolith inventory of soils in central Africa —quantities, shapes, classification, and spectra. *Review of Palaeobotany and Palynology* **107**, 23-53, doi:<http://dx.doi.org/10.1016/S0034-6667(99)00018-4> (1999).

15 Runge, F. & Fimbel, R. in *International Union for Quaternary Research. Proceeding of the VXth INQUA Conference (15 th: 1999).* 171-185 (Taylor&Francis, Tokyo).

16 Strömberg, C. A. E., Werdelin, L., Friis, E. M. & Saraç, G. The spread of grass-dominated habitats in Turkey and surrounding areas during the Cenozoic: Phytolith evidence. *Palaeogeography, Palaeoclimatology, Palaeoecology* **250**, 18-49, doi:<http://dx.doi.org/10.1016/j.palaeo.2007.02.012> (2007).

17 Aleman, J. C., Canal-Subitani, S., Favier, C. & Bremond, L. Influence of the local environment on lacustrine sedimentary phytolith records. *Palaeogeography, Palaeoclimatology, Palaeoecology* **414**, 273-283, doi:<http://dx.doi.org/10.1016/j.palaeo.2014.08.030> (2014).

18 Alexandre, A., Meunier, J. D., Lézine, A. M., Vincens, A. & Schwartz, D. Phytoliths: indicators of grassland dynamics during the late Holocene in intertropical Africa. *Palaeogeography, Palaeoclimatology, Palaeoecology* **136**, 213-229, doi:<http://dx.doi.org/10.1016/S0031-0182(97)00089-8> (1997).

19 Barboni, D., Bonnefille, R., Alexandre, A. & Meunier, J. D. Phytoliths as paleoenvironmental indicators, West Side Middle Awash Valley, Ethiopia. *Palaeogeography, Palaeoclimatology, Palaeoecology* **152**, 87-100, doi:<http://dx.doi.org/10.1016/S0031-0182(99)00045-0> (1999).

20 Diester-Haass, L., Schrader, H. & Thiede, J. Sedimentological and paleoclimatological investigations of two pelagic ooze cores off Cape Barbas, North-West Africa. *Meteor Forsch-Ergebnisse* **16**, 19-66 (1973).

21 Fredlund, G. G. & Tieszen, L. L. Calibrating grass phytolith assemblages in climatic terms: Application to late Pleistocene assemblages from Kansas and Nebraska. *Palaeogeography, Palaeoclimatology, Palaeoecology* **136**, 199-211, doi:<http://dx.doi.org/10.1016/S0031-0182(97)00040-0> (1997).

22 Bremond, L., Alexandre, A., Hély, C. & Guiot, J. A phytolith index as a proxy of tree cover density in tropical areas: calibration with Leaf Area Index along a forest–savanna transect in southeastern Cameroon. *Global and Planetary Change* **45**, 277-293, doi:<http://dx.doi.org/10.1016/j.gloplacha.2004.09.002> (2005).

23 Bremond, L. *et al.* Phytolith indices as proxies of grass subfamilies on East African tropical mountains. *Global and Planetary Change* **61**, 209-224, doi:<http://dx.doi.org/10.1016/j.gloplacha.2007.08.016> (2008).

24 Messager, E., Lordkipanidze, D., Delhon, C. & Ferring, C. Palaeoecological implications of the Lower Pleistocene phytolith record from the Dmanisi Site (Georgia). *Palaeogeography, Palaeoclimatology, Palaeoecology* **288**, 1-13 (2010).

25 Dey, S. B., Ghosh, R., Shekhar, M., Mukherjee, B. & Bera, S. What drives elevational pattern of phytolith diversity in *Thysanolaena maxima* (Roxb.) O. Ktze? A study from the Darjeeling Himalayas. *Flora-Morphology, Distribution, Functional Ecology of Plants* **211**, 51-61 (2015).

26 Issaharou-Matchi, I. *et al.* Intraspecific biogenic silica variations in the grass species Pennisetum pedicellatum along an evapotranspiration gradient in South Niger. *Flora-Morphology, Distribution, Functional Ecology of Plants* **220**, 84-93 (2016).

27 Twiss, P. C. in *Quaternary environments of Kansas* 179-188 (Kansas Geological Survey Guidebook Series 5, Lawrence, 1987).
