# Supplementary material for: Messinian age and savannah environment of the possible hominin Graecopithecus from Europe
Source: PLoS One. 2017 May 22;12(5):e0177347. doi: 10.1371/journal.pone.0177347 (PMC5439672; doi:10.1371/journal.pone.0177347)
Supplement: S4 Text — (DOCX) [file pone.0177347.s020.docx]

**Sedimentology**

Outcrops of the Pikermi Formation have been studied by us along a 700 m long transect (type section; N 30˚ 0.5’, E 23˚ 56.3’ to N 30˚ 0.3, E 23˚ 56.8’) of the ravine of the Megálo Réma rivulet directly north of Pikermi (Figure 1‒figure supplement 1a) and 1.6 km northeast in the former clay pit Chomateri (= Chomaterés, Kisdari hill). Sediments dip gently (2-3˚) towards southeast. Exposed sections along Megálo Réma are up to 4 m high and the river slopes slightly less (1.7˚) than sediment dip, so that a nearly continuous section could be measured between spots PV3 and PV1 (see also Fig 2).

The lower *Red Conglomeratic Member* (type section; N 30˚ 0.5’, E 23˚ 56.3’) is over 20 m thick (its base upslope of PV3 is obscured in the field) and consists of red (dry: hue 10R-5YR, chroma 6-8) silts, regularly subdivided by distinct para-conglomeratic beds. These beds represent lenticular shaped sheets with an observed lateral extension of several tens of meters. They have an erosional (scoured) base and are channelized at their deepest point. These up to 1.5 m deep channels are bordered by usually 0.3 m thick and over 10 m wide conglomeratic levees. Conglomerates are carbonate cemented and matrix-supported and composed exclusively by metamorphic clasts (dominantly mica-shists, rarely marbles) of the Attica Unit. Single clasts reach 20 cm in diameter and are well rounded. Conglomerate sheets show an indistinct inverse bedding (large cobbles on top) and their top can by highly irregular with an undulating surface (height differences up to 0.3 m) and projecting cobbles (Figure 2‒figure supplement 1d). Their matrix consists of middle to coarse sand. When several conglomerate beds are found in superposition (e.g. sub-sections PV3, Chom A) an alternation of dominant and faint sheets (identified in channelling depth and maximum grain size) is observable. The red clastics are dominated by silt (55-81%) and contain a various amount of clay (5-32%) and fine-sand (6-40%) fractions. Floating pebbles up to 3 cm in diameter can occur especially in association with bone accumulations. The red silts, interpreted as entisols, show no primary or secondary sedimentary structures, lack horizonation or pronounced pedogenic features and contain up to 67% carbonate (usually 30-40%), which occurs as microcrystalline impregnations and fine calcite nodules with diffuse boundaries. Sub-millimeter sized sparitic root tubules are frequent, whereas macrorizoliths are rare. Sediments of the Red Conglomeratic Mm have been sampled along the Megálo Réma at profiles PV 1 (= Pikermi Valley-1, new excavation led by George E. Theodorou; Theodorou et al. 2010), PV 3 (representing the classical site of excavations of Albert Gaudry 1855-1856 and 1860, A. Smith Woodward and Theodor Skoufos 1901, and Othenio Abel and Theodor Skoufos 1911-1912), and in the northern rim of the former clay pit Chomateri (lower part of section Chom A, directly above the excavations led by Nikolaus Symeonidis and Friedrich Bachmayer 1979-1980; ^1^).

The *Chomateri Member* (type section Chom B; N 30˚ 0.8, E 23˚ 57.8’)*,* which is best exposed on top of section Chom A and 80 m south in the type section Chom B (rim of Chomateri clay pit, including the level of excavations led by Nikolaus Symeonidis and Friedrich Bachmayer 1972-1978 ^2^)*,* is 7 m thick and characterized by reddish to yellowish (hue 5YR-7.5Y) silts, alluvial fine to middle sands and fluvial channels filled by medium to coarse sand with a minor content of pebbles (up to 4 cm in diameter). Individual fluvial bodies are interlocked to continuous laterally extensive channel-fill trains (Figure 2‒figure supplement 1f, g). The up to over 1 m thick channel-fills are trough cross-stratified, whereas 0.1-0.2 m thick crevasse splay bodies show no internal structures. Three successive fluvial episodes can be distinguished, of which the middle one is less distinctive (Figs 3e, 3f, and 3h). Palaeosols are strongly developed lateral to fluvial channels. The base of the member in section Chom B is characterized by a prominent 2.2 m thick calcic palaeosol with distinct E-Btk-Bk soil horizons (Fig 3h).

It is important to note that the lithologic characters of the Pikermi Formation, especially of the lower Red Conglomeratic Mb, are very similar to the local Pleistocene cover (Fig 2a) and difficult to distinguished in the field ^3-5^, which caused some misinterpretations in the past ^6-8^. Important differences are that Pleistocene debris flows volumetric dominate over fine-grained sediments, contains much larger marble clasts (up to 100 cm in diameter), are less cemented, and intercalated sediments contains no vertebrate fossils. Vertebrate fossils, which make Pikermi one of the most famous fossil localities in the world, appear in both members of the Pikermi Formation in at least eight horizons. Fossils, predominantly mammals, occur as densely packed bone beds, which can reach a maximum thickness of over 30 cm.

In present days the *Athens basin* is largely overbuilt by urban structures of the rapidly growing capital of Greece, so that our knowledge of Neogene sediments relies largely on the mapping and observations of Bruno von Freyberg ^9,10^. Freyberg ^10^ divides the Neogene into three units: a ‘lower limestone unit’ with grey marls and coals and abundant early Tortonian melanopsid gastropods ^11^, a ‘conglomeratic-sandy unit’ containing the *Graecopithecus*-bearing fauna of Pyrgos (= Pyrgos Vassilissis Amalias, Tour la Reine), and the ‘upper limestone unit’ with clays and platy white limestones, but without documented fossils. The ‘lower limestone unit’, with formerly extensive underground coal-mining activities, is heavily tectonically disturbed ^10,12^ and separated by an angular unconformity from the younger ‘conglomeratic-sandy unit’, which is concordantly overlaid by the ‘upper limestone unit’. Based on lithologic characters both latter stratigraphic units of v. Freyberg correspond well with the Pikermi and Rafina Formations as defined here from the Mesogea Basin. The fossil locality of Pyrgos was discovered by v. Freyberg in June 1944 in the course of gallery constructions tunnelled in a small hill 300 m southeast of the ‘Queens tower’ (Fig 2b). The underlying ‘lower limestone unit’ was drilled in a well at the ‘Queens tower’ ^10^. The accessible profile ^9^ at the fossil locality revealed 2 m of reddish to yellowish fine-sandy silt overlain by 2 m of reddish silt containing occasionally small pebbles. Both units are separated by laterally discontinuous packages of pebbly conglomerates (probably debris-flows). These sediments dip 12˚ toward east to south-east. The top of the section is built by at least 1.8 m thick conglomerates consists of cobble-sized components. Mammalian fossils have been found directly below this conglomerate in four horizontally discontinuous patches of bone accumulations. Nearly all fossils (n=49) are still embedded in their original matrix. The sediment is reddish (dry 10R-2.5YR 7/8) fine-sandy silt with few quartzite granules. There is no indication of clay illuviation and no carbonate nodules. Some large long-bones of giraffes are partially filled with sediment overgrown by geopedal sparry calcite, providing a way-up structure for palaeomagnetic analysis (S1 Fig).

For the Rafina Formation we not define formal members because of presently bad outcrop situations especially in the lower part due to excavations of clay and coal. The lower part is characterized by greyish to yellowish clays, intercalated by several dark organic rich clay horizons (not outcropping today because of total excavation in Chomateri; but see ^13-15^. These sediments contain (directly above the contact with the Chomateri Mb) a freshwater gastropod assemblage with *Planorbis*, *Theodoxus*, and *Melanopsis* ^14^, as well as a rather diverse small mammal fauna ^16-18^. The top of the lower part is characterized by two up to 2 m thick xylitic lignite beds and tree stumps in autochthon (upright) position ^13^. These coals have been excavated by the Rafina Lignite Mine ^12^.

The upper part of the Rafina Formation consists of >20 m thick alternation of whitish platy (5-10 cm thick) limestones and beige marls ^13^ containing lacustrine gastropods (Planorbidae), intercalated by thin organic rich layers (Figs 3i and 3j). The top of the upper part is composed of >1 m compact whitish fine silt, overlain by about 2 m thick double alternation of greyish platy fine-sandstones (0.5 m and 0.6 m thick), whitish fine silts (0.1 m and 0.08 m thick), and conglomeratic beds (each 0.4 m thick). Fine-sandstone plates show symmetric wave ripples on their top. The conglomerates are matrix (fine sand) supported and composed of well-rounded marbles (maximum diameter 15 cm). Outcrops of the upper part of the Rafina Formation are scattered between urban structures around 600 m east of Chomateri and at the left side along the street from Pikermi to Marathon and Rafina. Whereas sediments of the coal-bearing lower part dip gently (2-3˚) towards east and south ^12,13^ the upper lacustrine carbonates are disturbed by local faults and dip steeper with 5-10˚ in eastern to south-eastern directions (Fig 2a).

**References**

1 Bachmayer, F., Symeonidis, N. & Zapfe, H. Die Ausgrabungen in Pikermi-Chomateri bei Athen. Eine Dokumentation. *Annalen des Naturhistorischen Museums in Wien. Serie A* **84**, 7-12 (1980).

2 Symeonidis, N., Bachmayer, F. & Zapfe, H. Ausgrabungen in Pikermi bei Athen, Griechenland. *Annalen des Naturhistorischen Museums in Wien* **77**, 125-132 (1973).

3 Abel, O. in *Anz. Kais. Akad. Wiss. Math. Nat.* Vol. 49 325-327 (1912).

4 Trikkalinos, J. Tektonische und paläogeographische Untersuchungen der nachtertiären Schichten Attikas. *Prakt. de l'Acad. d'Athenes* **10**, 447-457 (1935).

5 Marinos, G. & Symeonidis, N. Neue Fossilfundstellen und neue Ergebnisse aus Pikermi (Attika). *Bull. Geol. Soc. of Greece* **9**, 160-176 (1973).

6 Gaudry, A. *Animaux fossiles et géologie de l'Attique: 2: Atlas*. 474 pp (F. Savy 1862).

7 Lepsius, R. *Geologie von Attika*. 237 (D. Reimer, 1893).

8 Quade, J., Solounias, N. & Cerling, T. E. Stable isotopic evidence from paleosol carbonates and fossil teeth in Greece for forest or woodlands over the past 11 Ma. *Palaeogeography, Palaeoclimatology, Palaeoecology* **108**, 41-53 (1994).

9 Freyberg, B. v. Die Pikermifauna von Tour la Reine. *Annales Géologiques des Pays Helléniques* **3**, 7-10 (1949).

10 Freyberg, B. v. Das Neogen Gebiet nordwestlich Athen. *Annales Géologiques des Pays Helléniques* **3**, 65-86 (1950).

11 Papp, A. Zur Kenntnis neogener Süsswasserfaunen in Attika (Griechenland). *Annales Géologiques Pays Helléniques* **29**, 664-678 (1979).

12 Toenges, A. L., Crentz, W. L., Parks, B. C. & Abernethy, A. F. *Lignite in Greece*. 57 pp (United States Bureau of Mines, 1951).

13 Marinos, G. Geological reconnaissance of the Rafina lignite basin. *Geological Reconnaissance* **18**, 1-7 (1955).

14 Marinos, G. & Symeonidis, N. Neue Funde aus Pikermi (Attika, Griechenland) und eine allgemeine geologische Übersicht dieses paläontologischen Raumes. *Annales Géologiques des Pays Helléniques* **26**, 1-20 (1974).

15 Symeonidis N., Bachmayer, F. & H., Z. *Pikermi, Field Guide to the Neogene of Attica*. Vol. 33 A (Department of Geology & Paleontology University of Athens, 1979).

16 De Bruijn, H. Vallesian and Turolian Rodents from Biotia, Attika and Rhodes (Greece). I and II. *Proc. Kon. Nederl. Akad. van Wetensch., B* **79**, 361-384 (1976).

17 Rümke, C. Insectivora from Pikermi and Biodrak (Greece). *Proc. Kon. Nederl. Akad. van Wetensch., B* **79**, 256-270 (1976).

18 Lopez Martinez, N. Lagomorpha from the Turolian of Pikermi (Greece). *Proc. Kon. Nederl. Akad. van Wetensch*, 235-244 (1976).
