# Supplementary material for: Messinian age and savannah environment of the possible hominin Graecopithecus from Europe
Source: PLoS One. 2017 May 22;12(5):e0177347. doi: 10.1371/journal.pone.0177347 (PMC5439672; doi:10.1371/journal.pone.0177347)
Supplement: S5 Text — (DOCX) [file pone.0177347.s021.docx]

**The Pyrgos Vassilissis mammalian fauna**

The mammalian fauna from Pyrgos Vassilissis is composed of 11 species, the majority of which is unknown from classical Pikermi levels (Table 1).

Proboscidea and Carnivora: Apart from a proboscidean metapodial fragment (TE 115) and an upper carnassial of the hyaenid *Adcrocuta eximia*, which is one of the most common carnivores in Turolian faunas, most of the Pyrgos fauna consists of ungulates.

Rhinocerotidae: The Pyrgos collections include various heavily damaged rhinocerotid specimens. AMPG 10 is a left mandibular fragment preserving the dp2 and the mesial lobe of dp3 (the distal lobe of dp3 has been modeled with plaster in the past). The teeth are at an early stage of wear. Dp2 differs from *Ceratotherium neumayri* in the less developed paralophid and in the posterior valley being more opened lingually. On dp3, a small fossettid is formed by the fusion of the mesial and distal lobes of the paralophid. The trigonid lacks a mesial vertical groove on its labial wall, and lingually the trigonid basin is open and V-shaped. According to ^1^, these morphological features distinguish *C. neumayri* from the contemporaneous *Dihoplus pikermiensis*. Paraskevaidis ^2^ referred a left maxilla fragment preserving the DP1–DP4 as “*Rhinocerus orientalis*”. Since this specimen has not been found in the Paraskevaidis collection, our observations are based on the photograph provided by ^2^. The teeth appear to be relatively higher-crowned than in Pikermi specimens of *D. pikermiensis* at the same stage of wear, and seem closer to *C. neumayri*. The postfossette of DP2 is very small. On DP3 the crista and crochet are present and form a closed medifossette. On the contrary, in *D. pikermiensis* the postfossette on DP2 is larger, and on DP3 the crista is absent and no medifossette is formed ^1,3^. Thus, this specimen seems closer to *C. neumayri*. A fragmentary left astragalus (TE 105) has a proximodistally much elongated (suboval) sustentacular calcaneal facet. Additionally, at its lateral surface the long, narrow facet for the fibula narrows proximally, and the area for the lateral talocalcaneal ligament is large. All these characters fit better *C. neumayri ^1^*, but the trochlea seems deeper/asymmetrical than the one in the *C. neumayri* sample from Pikermi. The few rhino materials from Pyrgos does not allow for definite taxonomic conclusions. However, specimens have similarities to *C. neumayri* and are herein referred to as ?*C. neumayri*.  Some morphologic disparities could indicate biochronologic difference of the Pyrgos population from Pikermi.

Equidae: The hipparion sample from Pyrgos is small and poorly preserved, but provides useful information. It consists of a cranial fragment, some upper teeth, mandibular fragments and a proximal third metacarpal. The better preserved of these is a cranial fragment (AMPG 02; Figs 7a and 7b, S6 Table) preserving both cheek toothrows, but missing its muzzle and its braincase. It is of medium size (distance orbit - P2 = 157.0 mm). Only the part above the cheek-teeth is preserved. The front border of the orbit is located a few millimeters behind the level of M3. The right preorbital bar is 58.3 mm broad. The preorbital fossa (POF) is small, oval, anteroventrally oriented, and of medium depth. Its posterior rim is well-marked, with a shallow pocket. The anterior rim is less distinct. The infra-orbital foramen is large, located in front of the POF, and opens anteriorly. The facial crest extends anteriorly as far as the level of the P4 metacone. The teeth are heavily worn, the protocone on P2 and M1 being connected to the protoloph. The advanced stage of wear and slight dislocation of the teeth result in a relatively short tooth row length (P2–M3 = 136.5 mm). The enamel plication is high (16 to 23 plis, despite the advanced wear). The pli caballin is double, but single on M1-2. The protocone is elongated, labially flattened. The hypocone is rounded. The hypoconal grove is shallow; there is a lingual groove on M3. Two other upper teeth (P4 and M1?) are less worn, and the enamel is more plicated (22 and 26 plis), the plis are deep and narrow, the pli caballin is double. The remaining upper teeth are unworn and their enamel pattern cannot be observed. On a mandible preserving both cheek toothrows (AMPG 03; S6 and S7 Tables), the enamel on the preflexid and postflexid is plicated and crenulated, the metaconid is rounded, and the metastylid is rounded or square. The ectoflexid is shallow on the premolars, but deep on the molars, reaching the lingual flexid on m1, but not on m2 and m3. The size of the proximal metacarpal (W = 44; APD = 30 mm; TE 128) is within the range of *Hippotherium brachypus*.

All observed features of the cranial fragment AMPG 02 are similar to those of *Hippotherium*. The small POF and its shallow posterior pocket allow us to exclude from the comparison *Hippotherium* *primigenium*, a Vallesian species, whose POF is large, deep, and with deep posterior pocketing ^4,5^. A single, oval POF, located far from the orbit, and with inconspicuous anterior border, can be observed in *Hippotherium* *brachypus* and in two *Hipparion* species (*H*. *dietrichi* and *H*. *prostylum*). The main differences between *Hippotherium* *brachypus* and the above-mentioned *Hipparion* species are the wider preorbital bar in *Hippotherium*, the presence of a posterior pocket, and the higher enamel plication, even in advanced wear stage, with deep, narrow plis. Therefore, the Pyrgos hipparion can be confidently identified as *Hippotherium* *brachypus*, but it displays some peculiarities. The preorbital bar is among the widest known for this species. Only a specimen from Azmaka (AZM) has a similarly broad bar (pers. observations); it is slightly narrower in some specimens from Hadzhidimovo in Bulgaria (HD) ^6^ and Pikermi (PIK) ^7,8^. Other *Hippotherium* specimens in the Balkans have narrower preorbital bars ^9-11^. The distance between the orbit and P2 and the tooth row length are close to the lower end of the variation range for *H*. *brachypus*, but the shortness of the tooth-row might result from heavy wear and distortion, as two virtually unworn P2 and P3 are longer than those of the cranial fragment. The only unusual feature of the Pyrgos cranium is the small preorbital fossa. Although the size of the POF of *H*. *brachypus* is highly variable (at Hadjidimovo, POF length varies from 47.4 to 76.7 mm, and height from 30 to 45 mm). The Pyrgos specimen has the smallest POF of all recorded *H*. *brachypus* specimens (length 42 mm, height 26 mm). Its size is smallest even among the *Hipparion* samples on the Balkans. The small size of the Pyrgos specimen’s POF could be accepted as an extreme variation of this feature. Thus, although generally similar to *H. brachypus* from other Middle Turolian faunas of the Balkans, some characters of the Pyrgos form set it somewhat apart.

Giraffidae: A right mandibular fragment (AMPG 18) preserves the m1 and the m2 (S8 Table). The m2 was partly inside the mandibular ramus (just erupting) and is completely unworn. While the teeth are comparable in size to those of *Bohlinia attica*, they differ in their distinctly greater hypsodonty. A left maxillary fragment (AMPG 15) with the M2 and M3 is of the same size class (LM3 = 28.5 mm) and is also characterized by more hypsodont molars (height at the paracone of M3 = 19.9 mm), flatter labial walls and weaker styles than *B. attica*. An articulated tarsus (TE 121) also differs from *B. attica* in that it has only one articular facet between the cubonavicular and the metatarsus. There are some further differences in size between these postcranials, the smallest one being a distal metacarpal, the largest metatarsal fragments, but all could be referred to a single species. Compared with the forms present at Pikermi (and in several other Eastern Mediterranean sites), it is distinctly larger than *Palaeotragus rouenii*, a small, slender-limbed species, but smaller and less stoutly-built than the large *Helladotherium*. It dimensions roughly matches instead the medium-sized *Bohlinia attica*, a close relative of the modern giraffe, but the above-mentioned morphological differences rule out this identification. It corresponds better to a group centered on *Palaeotragus coelophrys*, a species defined at Maragheh (Iran), of which various relatives have been reported from many Eastern Mediterranean sites, or to a small *Samotherium*, the most common giraffid genus at Samos, but which are both absent from the Pikermi classical fauna. Species identification of this taxon that could be a large-sized *Palaeotragus* would be premature on these scrappy remains, but it does document a difference with the famous Pikermi site, as this giraffid it is certainly not present at Pikermi. However, the maxilla fragment illustrated by Paraskevaidis ^2^ clearly belongs to *B. attica*, because some of its premolars have bifurcated parastyle, which is typical of *B. attica* ^12-15^. So, *B. attica* was definitely present in Pyrgos as well.

Bovidae: The bovids are represented in both Pyrgos collections by four taxa: *Tragoportax macedoniensis*, *Tragoportax* sp., Bovidae gen. et sp. indet., and *Gazella* sp. The most important bovid specimen is AMPG 19a (Figs 7c-e, S9 and S10 Tables), which belongs to a female individual of *Tragoportax macedoniensis*. This specimen was extracted from a small fossiliferous block, originally figured by Paraskevaidis ^2^ as “*Gazella* sp.?”. It is a partially preserved skull, medio-laterally compressed and skewed, which also lacks most of the right pre-orbital region. The braincase is broken behind the parietal-frontal suture. It preserves the left tooth row, but the M1 and M2 are heavily damaged. The left horn-core is better preserved (preserved length = 87 mm), whereas the right one is more broken and transversally crushed. The maximal width of the braincase behind the horn-core bases measures about 61.5 mm. In lateral view and with the tooth row horizontal, the anterior margin of the orbits is slightly rostral relative to the M2/M3 contact. The infra-orbital foramen opens above the middle of P2. The supra-orbital foramen opens above the center of the orbit; it is small, rather far (about 36 mm) from the base of the horn-core, with which it is connected by a shallow groove. The inter-frontal suture is rostrally smooth and open, but becomes invisible and crest-like caudally between the horn-core pedicles. The frontal parts of the temporal ridges are well-marked, but the parietal ones are not preserved. The choanae are not completely preserved; however, they must have been more caudal than the pterygoid fossae. The horn-cores are not twisted, they have a smooth anterior keel but no posterior one, and their basal cross-section is sub-oval. The distance between the horn-core bases is estimated at about 36 mm and the maximal external width at their bases is about 80 mm. The upper tooth row is characterized by the long premolar series relative to the molar one (S9 Table). The P2 is elongated (LP2 = 15.1 mm, WP2 = 11.6 mm) and strongly bilobate lingually, with the disto-lingual lobe extending farther lingually than the mesio-lingual one. The P3 is also lingually bilobate with a projecting disto-lingual lobe; however, it is more symmetrical (LP3 = 13.6 mm, WP3 = 13.3 mm). The P4 is broader than long (LP4 = 12.4, WP4 = 15.0). The M1 and M2 are heavily damaged. The M3 (LM3 = 16.6 mm, WM3 = 16.0 mm) is slightly damaged and lacks its paracone wall. It bears lingually a faint entostyle. The skull and horn-core morphology of AMPG 19a as well as the dimensions of its horn-cores fully correspond with those of female individuals of *T. macedoniensis* from Dytiko ^16^ (S9 and S10 Tables). Another specimen (AMPG 23) is an isolated, severely damaged right horn-core. The basal cross-section of the horn-core is sub-triangular. There is an eroded but clear anterior keel and, at the base of the horn-core, a trace of a postero-lateral keel. This specimen is poorly preserved. However, the horn-core morphology indicates that it could belong to a male individual of *T. macedoniensis*. Due to damage and distortion, the basal dimensions of the horn-core are approximate (APD = 38.0 mm, TD = 24.0 mm); nevertheless, they seem only slightly smaller than those of male individuals of *T. macedoniensis* from Dytiko localities (namely DTK, DIT, DKO; type locality DTK) in northern Greece. The generic status of this taxon is debated ^17-19^. However, our recent study (NS and SR) of the material from the type locality, stored in the University of Thessaloniki, has led us to the conclusion that this species belongs to the genus *Tragoportax*.

AMPG 17 is a right mandibular fragment preserving the m2 and the m3; the m3 lacks its third lobe. This specimen is comparable in tooth size and hypsodonty to a large *Tragoportax* (e.g. *T. amalthea*), but it is inadequately preserved; hence, it is referred to as *Tragoportax* sp. AMPG 16 is a right mandibular fragment preserving the distal lobe of m2 and the m3, and is herein attributed to Bovidae gen. et sp. indet. The m3 is completely unworn, large (occlusal length = 36.1 mm; basal length = 38.4 mm) and especially hypsodont (lingual height at the first lobe = 28.9 mm). It has a small ectostylid between the first and second lobes, and a faint one between the second and third lobes. The cross-section of its third lobe is rounded. Dimensionally, such an m3 size class has rarely been recorded in the late Miocene eastern Mediterranean mammalian faunas. In occlusal length, the m3 of AMPG 16 surpasses specimens of *Palaeoryx major* and *Palaeoryx pallasi* from Samos ^20^, specimens from Dytiko-2 (DIT) attributed to *Palaeoryx* sp. by ^19^ and specimens from Perivolaki attributed to *Palaeoryx* aff. *pallasi* by ^18^, and even specimens from Maragheh attributed by ^21^ to *Samokeros minotaurus*. From an ecological point of view, AMPG 16 represents a true grazer, contrary to *T. macedoniensis* which could be mostly a browser.

A frontlet preserving the bases of the horn-cores (TE 109) belongs to a medium-sized *Gazella*. Its features fit within the variation of the Pikermi *Gazella* sample. However, it could as well belong to another species; therefore it is referred to as *Gazella* sp.

**References**

1 Giaourtsakis, I. X. The Late Miocene mammal faunas of the Mytilinii Basin, Samos Island, Greece: New collection. 9. Rhinocerotidae. *Beitr. Paläont.* **31**, 157 (2009).

2 Paraskevaidis, E. in *VI Colloquium on the Geology of the Aegean Region, Proceedings.* 1143-1154 (Institute of Geological and Mining Research).

3 Geraads, D. Révision des Rhinocerotidae (Mammalia) du Turolien de Pikermi. Comparaison avec les formes voisines. *Ann. Paléont.* **74**, 13-41 (1988).

4 Bernor, R. L., Koufos, G., Woodburne, M. & Fortelius, M. The evolutionary history and biochronology of European and Southwest Asian Late Miocene and Pliocene hipparionine horses. *The evolution of Western Eurasian Neogene mammal faunas*, 307-338 (1996).

5 Bernor, R. L., Tobien, H., Hayek, L. A. C. & Mittmann, H. W. *Hippotherium primigenium* (Equidae, Mammalia) from the late Miocene of Höwenegg (Hegau, Germany). *Andrias* **10**, 1-230 (1997).

6 Hristova, L., Kovachev, D. & Spassov, N. *Hipparion brachypus* Hensel, 1862 from Hadjidimovo, Southwestern Bulgaria (Late Miocene). *Comptes rendus de l'Academie bulgare des Sciences* **56**, 2: 77 (2003).

7 Koufos, G. Study of the Pikermi Hipparions. Part I: Generalities and taxonomy. *Bulletin du Muséum national d'Histoire naturelle C* **9**, 197-252 (1987a).

8 Koufos, G. Study of the Pikermi hipparions. Part II: Comparisons and odontograms. . *Bulletin du Muséum national d'Histoire naturelle C* **9**, 327-363 (1987b).

9 Hristova, L. & Kovachev, D. in *Proceedings of the International Scientific conference (8-11.06. 2005). Faculty of Mathematics and natural sciences. South-Western University, Blagoevgrad.* 180-184.

10 Koufos, G. D. & Vlachou, T. D. Equidae (Mammalia, Perissodactyla) from the late Miocene of Akkaşdağı, Turkey. *Geodiversitas* **27** (2005).

11 Spassov, N. *et al.* The late Miocene mammal faunas of the Republic of Macedonia (FYROM). *Palaeontographica A* (in press).

12 Geraads, D. *Les Giraffidés du Miocène supérieur de la région de Thessalonique (Grèce)*, Université de Paris, (1974).

13 Geraads, D. Les Giraffinae (Artiodactyla, Mammalia) du miocène supérieur de la région de Thessalonique (Grèce). *Bulletin du Muséum national d’Histoire naturelle* **1**, 377-389 (1979).

14 Geraads, D., Spassov, N. & Kovachev, D. Giraffidae (Artiodactyla, Mammalia) from the Late Miocene of Kalimantsi and Hadjidimovo, Southwestern Bulgaria. *Geologica Balcanica* **35**, 11-18 (2005).

15 Geraads, D. Giraffidae (Mammalia) de la fin du Néogène de la République de Macédoine (ARYM). *Geodiversitas* **31**, 893-908 (2009).

16 Bouvrain, G. Les *Tragoportax* (Bovidae, Mammalia) des gisements du Miocène supérieur de Dytiko (Macédoine, Grèce). *Ann. Paléont.* **74**, 43-63 (1988).

17 Spassov, N. & Geraads, D. *Tragoportax* PILGRIM, 1937 and *Miotragocerus* STROMER, 1928 (Mammalia, Bovidae) from the Turolian of Hadjidimovo, Bulgaria, and a revision of the late Miocene Mediterranean Boselaphini. *Geodiversitas* **26**, 339-370 (2004).

18 Kostopoulos, D. S. Greek bovids through time. *Hellenic Journal of Geosciences* **41**, 141-152 (2006).

19 Bouvrain, G. & de Bonis, L. Ruminants (Mammalia, Artiodactyla : Tragulidae, Cervidae, Bovidae) des gisements du Miocène supérieur (Turolien) de Dytiko (Grèce). *Annales de Paléontologie* **93**, 121-147, doi:<http://dx.doi.org/10.1016/j.annpal.2007.03.001> (2007).

20 Kostopoulos, D. S. The late Miocene mammal faunas of the Mytilinii basin, Samos Island, Greece: new collection. 14. Bovidae. *Beiträge zur Paläontologie* **31**, 345-389 (2009).

21 Kostopoulos, D. S. & Bernor, R. L. The Maragheh bovids (Mammalia, Artiodactyla): systematic revision and biostratigraphic-zoogeographic interpretation. *Geodiversitas* **33**, 649-708 (2011).
